# Multi-platform quantitation of alpha-synuclein human brain proteoforms suggests disease-specific biochemical profiles of synucleinopathies

**DOI:** 10.1186/s40478-022-01382-z

**Published:** 2022-06-03

**Authors:** Tim E. Moors, Daniel Mona, Stefan Luehe, Gonzalo Duran-Pacheco, Liz Spycher, Olaf Mundigl, Klaus Kaluza, Sylwia Huber, Melanie N. Hug, Thomas Kremer, Mirko Ritter, Sebastian Dziadek, Gregor Dernick, Wilma D. J. van de Berg, Markus Britschgi

**Affiliations:** 1 Section Clinical Neuroanatomy and Biobanking, Department of Anatomy and Neurosciences, Amsterdam Neuroscience, Amsterdam UMC, Vrije University Amsterdam, Boelelaan 1108, 1081HZ Amsterdam, The Netherlands; 2 Ann Romney Center for Neurologic Diseases, Department of Neurology, Brigham and Women’s Hospital, Harvard Medical School, 60 Fenwood Rd, MA 02115 Boston, USA; 3 Roche Pharma Research and Early Development, Neuroscience and Rare Diseases Discovery and Translational Area, Roche Innovation Center Basel, Grenzacherstrasse 124, 4070 Basel, Switzerland; 4 Roche Pharma Research and Early Development, Therapeutic Modalities, Roche Innovation Center Basel, Grenzacherstrasse 124, 4070 Basel, Switzerland; 5 Roche Pharma Research and Early Development; Pharmaceutical Sciences, Biostatistics, Roche Innovation Center Basel, Grenzacherstrasse 124, 4070 Basel, Switzerland; 6 Roche Pharma Research and Early Development, Therapeutic Modalities; Large Molecule Research, Roche Innovation Center Munich, Nonnenwald 2, 82377 Penzberg, Germany; 7 Research & Development Roche Diagnostics Solutions, Roche Diagnostics GmbH, Nonnenwald 2, 82377 Penzberg, Germany; 8 Roche Pharma Research and Early Development; Oncology Discovery and Translational Area, Roche Innovation Center Basel, Grenzacherstrasse 124, 4070 Basel, Switzerland

## Abstract

Based on immunostainings and biochemical analyses, certain post-translationally modified alpha-synuclein (aSyn) variants, including C-terminally truncated (CTT) and Serine-129 phosphorylated (pSer129) aSyn, are proposed to be involved in the pathogenesis of synucleinopathies such as Parkinson’s disease with (PDD) and without dementia (PD), dementia with Lewy bodies (DLB), and multiple system atrophy (MSA). However, quantitative information about aSyn proteoforms in the human brain in physiological and different pathological conditions is still limited. To address this, we generated sequential biochemical extracts of the substantia nigra, putamen and hippocampus from 28 donors diagnosed and neuropathologically-confirmed with different synucleinopathies (PD/PDD/DLB/MSA), as well as Alzheimer’s disease, progressive supranuclear palsy, and aged normal subjects. The tissue extracts were used to build a reverse phase array including 65 aSyn antibodies for detection. In this multiplex approach, we observed increased immunoreactivity in donors with synucleinopathies compared to controls in detergent-insoluble fractions, mainly for antibodies against CT aSyn and pSer129 aSyn. In addition, despite of the restricted sample size, clustering analysis suggested disease-specific immunoreactivity signatures in patient groups with different synucleinopathies. We aimed to validate and quantify these findings using newly developed immunoassays towards total, 119 and 122 CTT, and pSer129 aSyn. In line with previous studies, we found that synucleinopathies shared an enrichment of post-translationally modified aSyn in detergent-insoluble fractions compared to the other analyzed groups. Our measurements allowed for a quantitative separation of PDD/DLB patients from other synucleinopathies based on higher detergent-insoluble pSer129 aSyn concentrations in the hippocampus. In addition, we found that MSA stood out due to enrichment of CTT and pSer129 aSyn also in the detergent-soluble fraction of the SN and putamen. Together, our results achieved by multiplexed and quantitative immunoassay-based approaches in human brain extracts of a limited sample set point to disease-specific biochemical aSyn proteoform profiles in distinct neurodegenerative disorders.

**Supplementary Information:**

The online version contains supplementary material available at 10.1186/s40478-022-01382-z.

## Introduction

Pathological accumulation of the protein α-synuclein (aSyn) in neurons and glial cells characterizes a group of neurodegenerative disorders, including Parkinson’s disease (PD), dementia with Lewy bodies (DLB) and multiple system atrophy (MSA) [1, 2]. aSyn is an abundant protein in the brain with a proposed role in regulating membrane trafficking and vesicular transport in presynaptic terminals while other functions—intra- or extracellularly as well as systemically—are yet poorly understood [3]. Genetic variation in *SNCA,* the gene encoding aSyn, including mutations [4–9], common variants and polymorphisms [10–12], but also gene multiplications [13, 14] provide evidence that aSyn and possibly regulation of its expression or clearance are a key in the pathogenesis of synucleinopathies. While the link between genetic alteration of *SNCA* expression and aSyn protein levels is not entirely understood, biochemical studies in the postmortem brain of patients further demonstrated the relevance of conversion of aSyn to more insoluble and heavily post-translationally modified (PTM) species in synucleinopathies: in particular Serine 129 phosphorylated (pSer129) and C-terminally truncated (CTT) proteoforms of aSyn are consistently detected in detergent-insoluble fractions from donors with PD and DLB [15–19] In support of these findings, immunostainings with antibodies specifically directed against pSer129 and CTT aSyn revealed their presence in Lewy bodies (LBs), Lewy neurites (LN), and glial cytoplasmic inclusions (GCIs, e.g. in oligodendrocytes), the neuropathological hallmarks of PD/DLB and MSA, respectively [17, 18, 20–22]. Confocal and super-resolution microscopy of Lewy pathology using specific antibodies directed against these forms of aSyn demonstrated their abundance and orchestrated distribution in these structures and support a central role of these PTMs in LB formation in the human brain [20, 21].

While the presence and subcellular distribution of these aSyn proteoforms in pathological structures has thus been consistently observed by immunostainings, quantitative data of pSer129 and CTT aSyn proteoforms in different regions of the human brain in conditions of physiological aging and synucleinopathy remain sparse [23–27]. The development of novel quantitative and reproducible assays to create an inventory of aSyn species in the human brain under physiological and various pathological conditions is of key importance to ultimately define which specific variants are associated with the pathology of different synucleinopathies [28]. In addition, such quantitative measures of different aSyn proteoforms could be used for better disease modelling in silico, for the development of better translational in vitro and in vivo models and in humans to discover novel aSyn-related biomarkers for early (differential) diagnosis, predicting disease progression and therapeutic response.

Here, we aimed to gain more insight in the abundance and solubility of aSyn protein variants in the human brain in physiological and pathological conditions, by providing quantitative measures of aSyn proteoforms in differential biochemical tissue extracts of hippocampus, putamen and substantia nigra (SN) from a total of 28 clinically and neuropathologically well-defined individuals with a synucleinopathy or other neurodegenerative disorders and from aged controls. We first detected disease-specific immunoreactivity signatures in these samples semi-quantitatively by a reverse phase array (RPA)-based multiplex approach with 65 antibodies against various specific aSyn protein domains and PTMs. We then validated and expanded our findings by quantifying total aSyn, aSyn C-terminally truncated at residue 119 (119CTT) and residue 122 (122CTT) or pSer129 aSyn in novel immunoassays with highly specific and well-characterized antibodies [21]. Our data suggest differential biochemical aSyn proteoform signatures between neurodegenerative diseases, and specifically within synucleinopathies.

Together, this comprehensive analysis of aSyn proteoforms in well-characterized patient samples extends the knowledge about abundance of aSyn in the human brain under conditions of normal aging and neurodegenerative disease such as synucleinopathy, and may contribute to a more quantitative aSyn-related pathological profiling of synucleinopathies.

## Methods

### Patient cohort and neuropathological staging

Fresh-frozen tissue of different brain regions (midbrain containing the SN, hippocampus -also containing part of the parahippocampal gyrus-, and putamen) was collected from donors with clinically diagnosed and neuropathologically confirmed PD (N = 5), PDD (N = 5), DLB (N = 5), MSA (N = 3), progressive supranuclear palsy (PSP; N = 2) and Alzheimer’s disease (AD; N = 3), as well as from age-matched neurologically normal subjects (hereafter referred to as ‘controls’, N = 5) (Table 1). For included MSA patients, clinical disease course and neuropathology were in correspondence with MSA-P. Postmortem brain tissue was collected by the Netherlands Brain Bank (www.brainbank.nl) in compliance with local ethical and legal guidelines and approved by the VU University Medical center ethics committee. Informed consent for brain autopsy and the use of brain tissue and clinical information for scientific research was given by either the donor or the next of kin. Brains were dissected in compliance with standard operating protocols of the Netherlands Brain Bank and BrainNet Europe [29]. Clinical information was requested from the treating physicians at the time of autopsy and summarized by an experienced assessor blinded to the neuropathological diagnosis, while the neuropathology was assessed by an experienced neuropathologist, according to the guidelines of BrainNet Europe [30, 31]. For the groups of patients with PD, PDD, DLB or MSA, we selected donors with limited concomitant AD pathology (Braak neurofibrillary tangle stage ≤ 3 and CERAD ≤ B) and without microinfarcts. Further, all controls, AD or PSP patients were devoid of LB pathology (Braak LB stage 0) [32]. Postmortem delay (PMD) for all donors was less than 10 h.

**Table 1:** Clinicopathological characteristics of donors included in this study

	Controls	PSP	AD	PD	PDD	DLB	MSA
Number	5	2	3	5	5	5	3
Age of death (mean ± SD)	80 ± 7	75 ± 2	80 ± 3	81 ± 6	81 ± 7	83 ± 4	66 ± 6*
M/F	2/3	2/0	0/3	3/2	4/1	4/1	1/2
Age of onset (mean ± SD)	n.a	68 ± 2	70 ± 4	70 ± 7	70 ± 6	75 ± 4	58 ± 5
Disease duration (mean ± SD)	n.a	6 ± 4	10 ± 7	11 ± 5	10 ± 3	9 ± 4	8 ± 3
Time to onset dementia (mean ± SD)	n.a	n.a	0	n.a	6 ± 2	0 ± 1	n.a
Postmortem delay in hours; median (range)	7 (6.4–7.6)	7.5 (5.3–9.8)	4.9 (4.0–6.1)	6.3 (5.2–7.4)	5.4 (4.0–7.1)	4.5 (4.0–6.0)	6.3 (4.9–6.8)
Braak NFT stage (median & range)	2 (1–3)	1 (1)	6 (5–6)	2 (1–2)	1 (1–2)	1 (0–3)	0 (0–1)
CERAD Amyloid Plaque score (median and range)	A (O-B)	A(A-B)	C (C)	O (O-A)	A (O-B)	B (O-B)	A (O-B)
Thal phase for Amyloid [93] (median & range)	1 (0–2)	1 (1)	4 (4–5)	0 (0–2)	2 (0–3)	3 (0–4)	1 (0–2)
Braak LB stage (median and range)	0 (0)	0 (0)	0 (0)	6 (4–6)	6 (4–6)	6 (6)	0 (0)
Brain regions analyzed	HIP, PUT, SN	HIP, PUT, SN	HIP, PUT	HIP, PUT, SN	HIP, PUT, SN	HIP, PUT, SN	HIP, PUT, SN

Pearson chi-square test was performed for comparisons between diagnostic groups; * *p* ≤ 0.05

*CERAD* Consortium to Establish a Registry for Alzheimer's Disease, *M* male, *F* female, *NFT* neurofibrillary tangles, *LB* Lewy body, *PSP* progressive supranuclear palsy, *AD* Alzheimer’s disease, *PD* Parkinson’s disease, *PDD* Parkinson’s disease with dementia, *DLB* dementia with Lewy bodies, *MSA* multiple system atrophy, *SD* standard deviation, *n.a.* not applicable, *HIP* hippocampus, *PUT* putamen, *SN* substantia nigra

### Brain tissue processing and fractionation

**Fig. 1:**
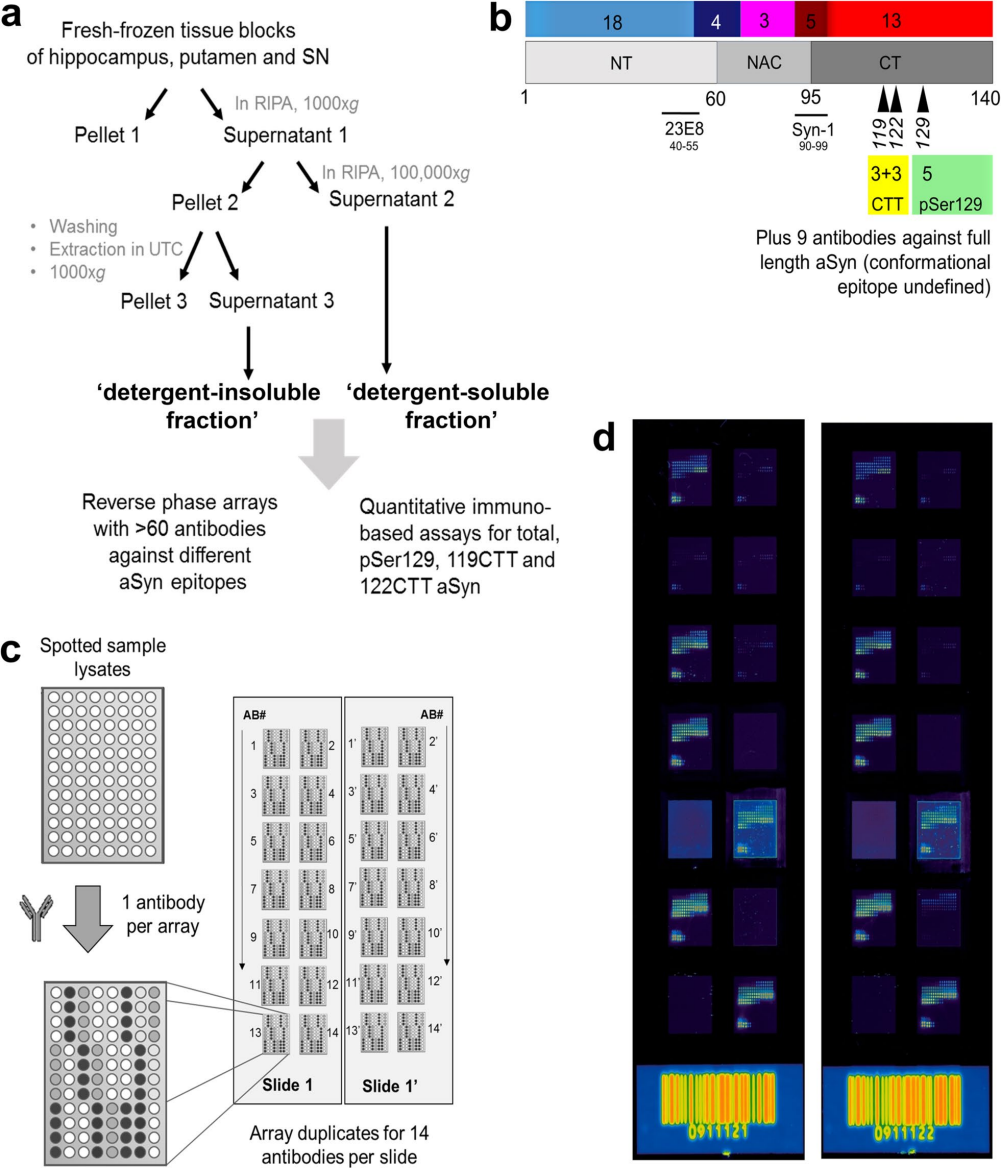
Tissue processing and reverse phase arrays (RPAs). **A** Workflow for tissue processing towards biochemical analysis of brain tissue extracts. For detailed description, see “Methods”. **B** Schematic of aSyn with number of antibodies per epitope group included in RPA analysis indicated above the schematic (see also Additional File 1: Table S2). 23E8 and Syn-1 antibodies were also used in alphaLISA® to measure ‘Total aSyn’. Arrow heads indicate epitopes for antibodies specific and selective against posttranslational modification 119CTT, 122CTT, and pSer129 and some were used together with 23E8 in alphaLISA® for the quantification of the respective forms of aSyn. **C** Workflow for RPAs: samples were spotted on nitrocellulose film slides. Pairs of pads were first incubated with each to generate two replicate incubations for all included antibodies, and afterwards with the species-matching fluorescently labeled secondary antibody. For detailed description, see “Methods”. **D** Example of two scanned slides containing duplicates for 14 antibodies in the analysis. Abbreviations: *RIPA*: radio-immunoprecipitation assay buffer containing protease and phosphatase inhibitors; *UTC*, extraction buffer containing urea, thiourea and 3-[(3-cholamidopropyl)dimethylammonio]-1-propanesulfonate hydrate (CHAPS); SN, substantia nigra

An overview of the workflow for tissue fractionation of the brain tissue samples is presented in Fig. 1A. Note, the order of processing the tissue was randomized by donor to avoid any technical bias and done separately per brain region. Frozen tissue blocks were manually sliced into sections in a cryostat and tissue (40–70 mg) was stored in Eppendorf tubes at − 80 °C until further processing. After quickly washing the tissue in PBS pH 7.2 (Gibco, 20012-019) containing 1 × complete protease inhibitor cocktail (Roche, 11 873 580 001) and 2 × phosphatase inhibitor cocktail set II (Calbiochem, 524,625) for thawing and blood removal, tissues were immediately transferred to MagNA Lyser tubes (Roche, 03 358 941 001, Basel, Switzerland) containing 1:10 (w/v) precooled 1 × sodium dodecyl sulfate (SDS)-free radio-immunoprecipitation assay (*RIPA*; Cell Signaling, 9806) buffer with 1 × complete protease inhibitor cocktail and 2 × phosphatase inhibitor cocktail set II. We employed a mild detergent mix lacking SDS (detergents according to manufacturer: 1% NP-40, 1% sodium deoxycholate; known for mostly cytoplasmic protein extraction leaving nuclear membranes intact) in order to avoid any unwanted interference of aSyn with SDS micelles and nuclear DNA [33–37]. Subsequently, tissue was homogenized using a Precellys homogenizer (6500 rpm, 1 × 20 s, ~ 5–10 °C in rotor compartment art. no: 03119.200.RD000; Bertin Technologies SAS, France) with Cryolis cooling unit (filled with dry ice, art.no 05068.200.RD000; Bertin Technologies). Between the two homogenization steps, the tubes were incubated on ice for 15 min, while the tubes were centrifuged for 2 min at 4 °C at 1000×*g* after each homogenization and were additionally shortly ventilated with ethanol vapor to reduce foam load after 2nd homogenization. Tissue homogenates were transferred (without ceramic beads) to 1.7 ml tubes (Sigma-Aldrich, art. no. T3406-250EA) and centrifuged for 10 min at 1000×*g* at 4 °C (Eppendorf Centrifuge, art. no. 5417R). The supernatant 1 (S1) was transferred in a centrifugal tube (Beckman, art. no. 357448; 1.5 ml-Polyallomer-Tubes) and centrifuged at 100,000×*g* at 4 °C for 30 min (Beckman TL-100 ultracentrifuge with rotor TLA-55). This *RIPA*-soluble fraction containing supernatant 2 (S2) will be further referred to in the text as detergent-soluble fraction. It was removed from pellet 2 (P2), aliquoted, and immediately frozen on dry-ice and stored at − 80 °C. P2 was immediately frozen on dry-ice where it was kept for at least 15 min or stored at − 80 °C as well. Subsequently, it was thawed on ice and washed by resuspending in 500 µl cold wash-buffer (= PBS + phosphatase inhibitors + protease inhibitors) after which it was centrifuged at 100,000×*g* at 4 °C for 30 min. This washing step was repeated once. The double washed P2 samples were diluted 1:1 in an extraction buffer (further referred to as *UTC*-buffer, containing 7 M urea (Merck, art. no. 1.08487.1000), 2 M thiourea (Sigma-Aldrich, art. no. 88810), 30 mM tris–CL pH 7.5 (Sigma, art. no. T5941), and 4% 3-[(3-Cholamidopropyl) dimethylammonio]-1-propanesulfonate hydrate (CHAPS; Sigma-Aldrich, art. no. C3023), and incubated 1 h at room temperature. The whole suspension was subsequently frozen again at -80 °C for at least 15 min, after which P2 samples were thawed to room temperature, vortexed and centrifuged 10 min at 1000×*g*. This *UTC*-soluble fraction containing supernatant 3 (S3, the P2 extract) will be further referred to in the text as detergent-insoluble fraction. It was removed from pellet 3 (P3), aliquoted, immediately frozen on dry-ice, and stored at − 80 °C, and so was P3. Content and quality of extracts were inspected for all samples by Coomassie blue and silver stainings of SDS PAGE electrophoresis gels. Some urea insoluble pellets P3 were boiled directly in SDS sample loading buffer for solubilization towards SDS-PAGE analysis. As these samples did not reveal a signal for aSyn by Western blot and the pellet was not compatible for the RPA or the sandwich immunoassays, P3 was not further analyzed in this study. Total protein concentrations of the detergent-soluble and detergent-insoluble fraction, respectively, were determined using by bicinchoninic acid (BCA) assays. For detergent-soluble fractions protein concentration was measured by a Pierce micro BCA kit (Thermo Scientific, art. no: 23235). For the insoluble fraction, the 660 nm BCA kit was used because of its compatibility with urea containing buffers (Thermo Scientific, art. no: 22662). All protein assays were designed and performed according to manufacturer’s guidelines. Back-calculation from mg/ml to mg/g wet volume was performed by dividing the BCA concentration with the weight per volume ratio (i.e., 1:10 mg/ml for the detergent-soluble and 1:1 mg/ml for the detergent-insoluble fraction).

### Generation of recombinant aSyn proteoforms

Recombinant human full length, 119CTT and 122CTT aSyn proteoforms were generated according to the protocol previously published by our and other groups [18, 38]. Polo like kinase 2 (PLK2) was expressed in BL21-DE3-pLysS competent *E. coli*, isolated via its His-tag and immediately used to phosphorylate purified aSyn. Briefly, aSyn was mixed with PLK2 buffer (200 mM Tris pH 7.5/100 mM MgCl2/10 mM DTT), 10 mM ATP, H_2_O and PLK2, in a molar ratio of aSyn to PLK2 of 167:1. After incubation for 24 h at 30 °C without shaking, samples were diluted 1:3 in 20 mM Tris pH 7.5 buffer (buffer A) and loaded on a 1 ml HiTrap Q column (GE Healthcare). After washing with buffer A, the proteins were eluted with a gradient from 20–55% buffer B (20 mM Tris pH 7.5/1 M NaCl) in 35 column volumes. Chromatography was performed on a ÄKTA explorer (GE Healthcare). The fractions containing phosphorylated aSyn were pooled, concentrated to 0.91 mg/ml (measured with Pierce micro BCA protein assay; Thermo Scientific) using a 3kD Amicon Ultra unit, aliquoted and frozen at − 80 °C.

### Generation and characterization of antibodies towards specific aSyn epitopes

A detailed overview for all utilized antibodies and their epitopes in this study is provided in Fig. 1B and Additional File 1: Table S2. Usage in immunostaining studies and additional characterization of antibodies Syn105, 11A5, 23E8, 5C1, syn-131 (119CTT aSyn), syn-134 (122CTT aSyn), and syn-142 (pSer129 aSyn) was previously published as well [18, 21, 39, 40]. Antibodies generated at Roche and Prothena were characterized and validated with highest industrial standards and very stringent exclusion criteria based on determination of specificity using biochemical assays and ability to immunoreact with Lewy pathology in postmortem human brain sections on fresh-frozen or paraffin embedded tissue. Briefly, at Roche, novel antibodies were generated by immunizing rabbits either with *E. coli* derived recombinant full length (aggregated) human aSyn, KLH-conjugated peptides or aSyn derived peptide phosphorylated at Ser129. All animal experiments followed highest animal welfare standards and were performed according to ethics protocols approved by the local animal welfare committee at Roche, while animal experiment licenses were approved by the respective state authorities. After screening of serum and B cell culture supernatant titers, standard B cell cloning was performed to generate rabbit monoclonal antibodies (mAbs) [41]. Recombinant mAbs were screened for binding to full length recombinant human aSyn, the aSyn peptides aa1-60 and aa96-140 or to aSyn119CTT, aSyn122CTT or phosphorylated at Ser129 by ELISA and surface plasmon resonance (SPR). Counter-screen by ELISA was performed with beta- and gamma-synuclein for mAbs generated in immunization campaigns against full length aSyn. For CTT and pSer129 aSyn specific antibodies, surface plasmon resonance (SPR) was employed using the mAbs captured by a Fc specific anti-rabbit polyclonal antibody on the sensor chip and biotinylated peptides corresponding to the C-termini of truncated a-synuclein (i.e. aSyn(aa 106–119) or aSyn(aa 106–122), respectively) or the peptide aSyn(122–135) phosphorylated at Ser129 as analytes, respectively. Biotinylated C-terminal elongated peptides (aSyn(aa 106–122) or aSyn(aa 106–124), respectively) or the un-phosphorylated peptide aSyn(122–135) have been used as negative controls to demonstrate the specificity of the mAbs for the truncated C-terminal sequence or pSer129, respectively. Roche antibodies generated in immunization campaigns against full length aSyn were further epitope mapped on custom made PepStarTM peptide microarrays (JPT Peptide Technologies, Berlin, Germany) following the manufacturers instruction. Peptide sequences had a length of 15 amino acid residues and were designed to cover the whole sequence of human aSyn. Neighboring peptides had an overlapping sequence of 11 amino acids. All antibodies were also positive for binding human recombinant aSyn on Western blot and labelled synaptic or cytosolic aSyn or Lewy pathology in fresh frozen brain sections from independent normal controls or PD cases, respectively. Description about generation and characterization of Prothena antibodies was published previously [18, 39, 40] and was also made available in a patent (WO 2007/021255 A1). Different commercially available antibodies were included in our assays as well: Syn-1 (epitope: 91–99, BD biosciences, art no: 610787), clone 211 (epitope: 121–125, Santa Cruz, art. no: 12767), 4B12 (Covance, art. no: #SIG-39730); 15G7 (epitope: 117–131, Enzo LifeSciences, art. no: ALX-804–258-L001) [21, 23, 42–45].

### Reverse phase array assays

Reverse phase protein arrays were generated with brain tissue extracts of all included donors [46]. The extracts were spotted contact free (Nanoplotter 2.1, GeSiM, Radeberg, Germany) on 16-pad nitrocellulose film slides (ONCYTE Avid, Grace Biolabs #305116, Bend, Oregon, United States). Spotted slides were placed in a NEXTERION® IC-16 (Schott Nexterion #1262705, Jena Germany) for blocking and antibody incubation. Slides were blocked for 15 min in a 1:2 dilution of Odyssey blocking buffer (art. no. 927-40000, Li-Cor Biosciences, Bad Homburg, Germany) and 0.05% Tween in PBS (art. no. 524653-1EA, Merck Millipore, Burlington, Massachusetts, United States). This buffer was also used as assay buffer for the dilution of primary and secondary antibodies. After the blocking buffer was removed, six pairs of pads were first incubated with each of the 65 in-house and commercial antibodies with different specificities towards amino acid sequences and regions of (PTM) aSyn (see above) to generate two replicate incubations per antibody (Fig. 1). In addition to antibodies against aSyn, we also used antibodies detecting the neuronal nuclear antigen (NeuN, also known as Fox-3; mostly located in the nuclear subcellular compartment in neurons), the pan-neuronal and cytoplasmic enzyme neurons-specific enolase (NSE), and the presynaptic vesicular membrane protein synaptophysin (for details about the antibodies see Additional File 1: Table S2) in order to get an understanding of the robustness of our fractionation protocol and the multiplexed tissue-extract array method. For the detection of the primary antibody, arrays were incubated with the species matching fluorescently labeled secondary antibody (Li-Cor #926-32210, #926-32211, and #925-32219). The slide was washed two times between primary and secondary antibody incubation with assay buffer. Primary antibodies were diluted 1:1000 and incubated for 18 h at RT. Secondary antibodies were diluted 1:10,000 and incubated for 1 h at RT. After secondary antibody incubation, the nitrocellulose-coated slide was washed two times with 0.05% Tween 20 in PBS for 10 s, finally rinsed with Milli-Q water and blow dried with nitrogen. Fluorescent signal intensities were measured by scanning the slides with an InnoScan 710 AL infrared microarray scanner (Innopsys, Carbonne, France) with 5 μm/pixel at 785 nm and PMT gain of from 20 to 100%. Raw spot intensities were extracted from images by the scanner software (Mapix, Innopsys, Carbonne, France) and processed as described in the statistical analysis section.

### Electrochemiluminescence-based immunoassay for the quantification of a total aSyn form

One quantification method for putative total aSyn (defined by an antibody pair that binds to N- and C-terminus, respectively and assuming binding of these antibodies is independent of modifications beyond their epitopes at N- or C-terminus), was performed using the Elecsys® Total aSyn Prototype Assay (Roche Diagnostics, Penzberg, Germany; proprietary, not commercially available). This method was previously established as research-grade electrochemiluminescence immunoassay for human CSF and serum and plasma [47]. Here, we adapted this assay using an in-house developed protocol to detect of aSyn proteoforms in brain extracts. For this purpose, all samples for detergent-soluble and detergent-insoluble fractions were 1:100 diluted in PBS buffer pH 7.4 and run on a Cobas® e411 immunoassay analyzer (Roche Diagnostics). Obtained concentrations were back-calculated from ng/ml to µg/g wet tissue.

### Design, optimization and execution of AlphaLISA® assays for different forms of aSyn

Additional immunoassays for putative total aSyn (e.g., forms of aSyn that contain the epitopes for 23E8 and Syn-1, aSyn regions 40–55 and 90–99, respectively; herein called ‘total aSyn’), 119CTT aSyn, 122CTT aSyn, and pSer129 aSyn were developed for a 384 well plate format (AlphaLISA® platform, Perkin Elmer) with a total assay volume of 50 µl per well. Assays were designed using an antibody directed against the NT of aSyn (res. 40–55; 23E8; Additional File 1: Tables S1, S2) which was linked with the donor beads by its biotinylation using an EZ-link Sulfo-NHS-Biotin labeling kit (Pierce, art. no. 21326) according to manufacturer’s instructions. The commercial antibody Syn-1 (res. 91–99; art. no. 610787, BD Biosciences, Oxford, UK) was coupled to the acceptor beads (art. no.: 6762001; Perkin Elmer) according to the manufacturer’s instructions for detecting Total aSyn, while the Roche developed and characterized antibodies specifically against pSer129 aSyn, 119CTT and 122CTT aSyn were coupled to acceptor beads for the specific measurement of these PTMs (Additional File 1: Table S1). For each antibody pair, a hook point was acquired and an immuno-assay for each aSyn form was developed according to the manufacturer’s instructions (Additional File 1: Fig. S1). To generate a standard curve the respective recombinant aSyn was diluted in AlphaLISA® buffer (Additional File 1: Fig. S1). Exact concentration of the full-length aSyn was previously determined by amino acid analysis (AAA)-mass spectrometry and served as standard for the recombinant 119CTT, 122CTT, and pSer129 aSyn in the BCA assay for total protein concentration these aSyn forms.

Optimal sample dilutions were determined for each immunoassay and separately for detergent-soluble and -insoluble fractions, in order to measure samples in the linear range of the standard curve and to avoid matrix effects. For the measurement of detergent-soluble and -insoluble total aSyn, samples were diluted 1:100 in AlphaLISA® buffer. For the measurement of detergent-soluble and -insoluble 119CTT, 122CTT and pSer129 samples were diluted 1:10 and 1:20, respectively. In each assay, 5 µl of diluted sample was added to the plate, and incubated with 10 µl biotinylated 23E8 and 10 µl acceptor bead-coupled antibodies at RT, on a shaking plate set at 600 rpm (total aSyn: 24 h; other aSyn forms: 4 h). Acceptor bead-coupled antibodies were used at a concentration of 10 µg/ml in the final well volume. After incubation, 25 µl streptavidin-coated donor beads (end concentration in well: 40 µg/ml) were added, the plate was covered with aluminum foil and incubated for 30 min at room temperature on a shaking plate set at 600 rpm. Detection was performed directly afterwards on EnVision Multilabel Plate Reader (art. no: 2103; Perkin Elmer) in AlphaScreen mode.

Every plate contained randomized samples for the entire cohort (all brain regions), together with a standard curve. All measurements were done in triplicates, median values were used for further analyses. Concentrations were calculated for all samples, using standard curves in combination with XLFit Software (ID Business Solution, Guildford, UK). For each assay, lower limit of detection (LLD) and lower limit of quantification LLOQ) were further calculated (Additional File 1: Fig. S1 and Table S1). When concentration for a particular form of aSyn was lower than the LLD, the value of the LLD was assigned and used for further statistical analyses. All concentrations were expressed as µg/g wet tissue. Obtained concentrations were back-calculated from ng/ml to µg/g wet tissue. In order to enable a more relative comparison between the levels of the PTM aSyn proteoforms with the levels of the putative total aSyn levels, we defined hypothetically, that the level of total aSyn in each fraction type (i.e. in the detergent-soluble and the detergent-insoluble fraction, respectively), is 100% of all aSyn present in that fraction and that the PTM aSyn levels are a certain percentage of it (i.e., concentration of the respective aSyn proteoform divided by the total aSyn concentration).

### Statistical analysis

For the analyses of both RPA and AlphaLISA® results, between-group comparisons were conducted on log2-transformed signal intensities and protein levels measured by RPA and AlphaLISA®, respectively, using linear models in R software [48] using custom-written scripts. Pairwise comparisons were done with Dunnett’s post-hoc tests. *P* values for the comparison of each group versus controls were log10-transformed and indicated as positive or negative based on the direction of the mean difference compared to controls (with positive *p* values in case of increased aSyn levels in diseased groups vs controls and vice-versa). Transformed *p* values were visualized using heat maps of unsupervised hierarchical cluster analyses using R software [48]. Nonparametric tests were also applied on untransformed immunoreactivity data to verify our results are robust to the fulfillment of assumptions of parametric methods on log transformed values.

## Results

### Description of cohort characteristics

To allow studying aSyn proteoforms in contexts of physiological aging, synucleinopathy and other neurodegenerative conditions, we selected a patient cohort with advanced and relatively ‘pure’ pathologies (e.g. patient groups with limited concomitant pathology). Demographics, clinical details and pathological stages of the donors included in this study are summarized in Table 1. The study cohort included donors with clinically diagnosed and pathologically confirmed PD with (PDD; N = 5) and without (PD; N = 5) dementia, DLB (N = 5), MSA (N = 3), progressive supranuclear palsy (PSP; N = 2) and Alzheimer’s disease (AD; N = 3), as well as age-matched neurologically normal subjects (hereafter referred to as ‘controls’, N = 5) (Table 1). Cases were carefully selected based on short postmortem interval (< 10 h) and on limited concomitant pathology. For all included donors, clinical diagnosis during life was confirmed after neuropathological examination. All included PD/PDD/DLB patients showed widespread LB pathology at autopsy (corresponding with Braak LB stage 6 [32]) while extensive GCI pathology was observed in MSA patients. Clinical course and pathology of all included MSA patients was in correspondence with a parkinsonian MSA subtype (MSA-P). All included donors with a synucleinopathy had limited concomitant AD pathology (Braak neurofibrillary tangle stage ≤ 3 and CERAD ≤ B) and no microinfarcts. Selected AD/PSP donors showed widespread amyloid-beta (AD) and/or tau (AD + PSP) pathology but were devoid of LB pathology (Braak LB stage 0), as were the controls. Diagnostic groups were age-matched with exception of MSA patients, which were significantly younger than the other groups (Table 1). Patient groups further showed differential gender distribution reflecting higher prevalence for males or females for different neurodegenerative conditions [49].

### Immunoreactivity profiles of aSyn antibodies on reverse-phase array of human brain extracts suggest differential biochemical aSyn profiles between synucleinopathies

Based on previously published results [18, 19, 24, 26, 50, 51] we hypothesized that different forms of aSyn in human brain segregate differentially by solubility in patients with synucleinopathies compared to control subjects and patients with other neurodegenerative conditions. In order to explore this, we generated consecutive *RIPA*-soluble (without SDS) and *RIPA*-insoluble fractions of two to three different brain regions per subject, henceforth called detergent-soluble and detergent-insoluble fractions, respectively (Table 1 and Fig. 1) [52]. The total protein levels over all samples (including different brain regions and diagnostic groups) within the respective extract types were similar, indicating that protein extraction efficacy was comparable between the different samples and brain regions (Additional File 1: Fig. S2A, mean ± SD values for hippocampus/putamen/SN: 48.3 ± 7.0, 51.4 ± 6.7, and 50.0 ± 7.0 mg/g wet tissue, respectively).

To allow for a multiplexed analysis of proteins in the detergent-soluble and -insoluble fractions, we employed the RPA method (Fig. 1). In order to explore the extraction efficiency with the mild SDS-free RIPA buffer we included antibodies against well-established neuronal proteins with known differential subcellular distributions, such as the neuronal nuclear marker NeuN, the pan-neuronal and cytoplasmic enzyme NSE, and the presynaptic vesicular membrane protein synaptophysin (Additional File 1: Table S2 and Fig. S2A). As anticipated, the NeuN signal was low in all samples from all brain regions in the detergent-soluble fraction and enriched in the detergent-insoluble fractions, indicating that contribution of the nuclear compartment to the detergent-soluble fraction was minimal. In contrast and as anticipated, the NSE segregated strongly to the detergent-soluble fraction, indicating an efficient extraction of highly soluble cytoplasmic proteins. Synaptophysin also showed a strong enrichment in the detergent-soluble fraction suggesting that the SDS-free RIPA buffer was capable of efficiently extracting proteins that are associated with vesicular membranes. The clear detection of these well-established neuronal markers on RPA and their segregation into the expected extracts supports the robustness of our fractionation protocol and the multiplexed tissue-extract array method.

**Fig. 2:**
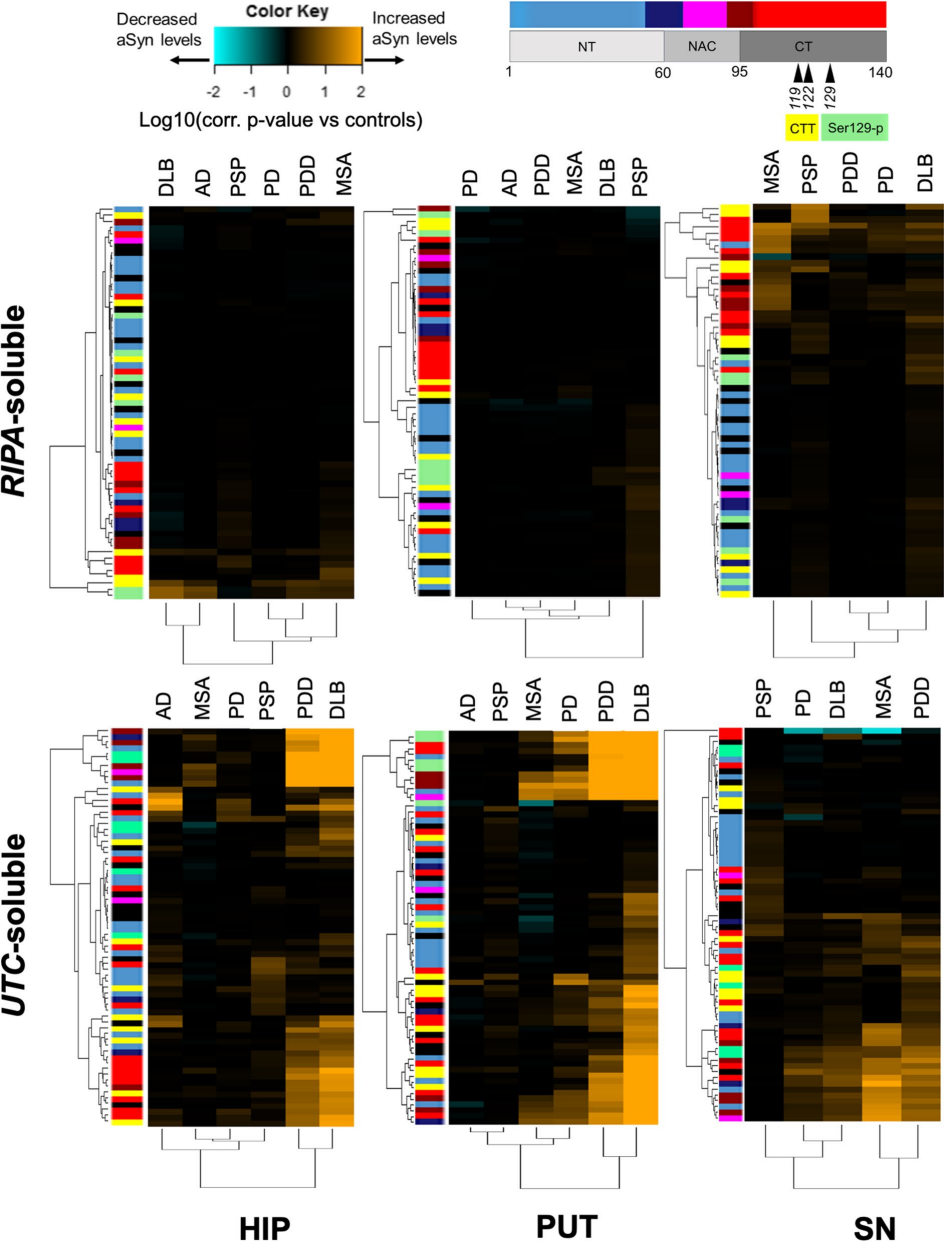
RPA immunoreactivity patterns towards fractionated brain extracts suggest disease-related biochemical aSyn manifestations in different neurological disorders. Heat maps of log10-transformed p-values of group comparisons in detergent-soluble and -insoluble fractions after Dunnett’s post-hoc correction. Colors indicate the significance level for each diagnostic group compared to neurologically normal subjects (controls), with orange indicating higher RPA immunoreactivities, cyan values indicating lower RPA immunoreactivities, while black indicates no difference (see also color key). Heat maps were generated by unsupervised hierarchical clustering. On top of each heat map, the clustering of antibody reactivities according to their epitopes is shown. HIP, hippocampus; PUT, putamen; SN, substantia nigra. Note: SN from AD was not extracted

We probed the RPA with 65 antibodies specific towards various aSyn protein domains and PTMs (Fig. 1 and Additional File 1: Table S2). The respective p-values that resulted from the comparison of the differential immunoreactivity between each diagnostic group versus controls was then employed for an unsupervised hierarchical clustering and heat map analysis (Fig. 2). The immunoreactivity pattern of aSyn proteoforms in the detergent-soluble fractions from the hippocampus and putamen was mostly comparable in groups with neurological diseases (both synucleinopathies and non-synucleinopathies) versus controls (Fig. 2). In contrast, various aSyn antibodies showed increased immunoreactivities in SN extracts of patients with a synucleinopathy compared to controls, particularly antibodies with epitopes at the CT or against CTT aSyn, while antibodies against NT aSyn and Ser129p aSyn showed similar reactivities between diagnostic groups (note: SN from AD was not extracted). Despite the limited number of samples in MSA, extracts from SN showed most marked differences versus controls, resulting in a separate clustering of the MSA patient group from the other neurological diseases (Fig. 2).

In contrast to the detergent-soluble fraction, detergent-insoluble tissue fractions showed strongly increased RPA immunoreactivities for many aSyn antibodies mainly in patients with synucleinopathies compared to controls (Fig. 2). This was most pronounced in the hippocampal/putamen extracts of PDD and DLB patients, resulting in a separate clustering of these patient groups from other neurological diseases. While detergent-insoluble SN extracts of PDD/DLB patients were as well enriched in aSyn immunoreactivity compared with controls, this increase was not as high in magnitude as the other two brain regions. Particularly in DLB patients, differences versus controls in the detergent-insoluble extracts of the SN were less pronounced compared to detergent-insoluble extracts of hippocampus/putamen, resulting in a clustering separate from PDD patients together with PD patients (Fig. 2). Immunoreactivity patters of MSA patients clustered together with those of PDD patients based on higher differences vs controls in SN extracts.

Overall, most prominent differences in immunoreactivity in detergent-insoluble fractions were observed for antibodies recognizing pSer129 aSyn and CT aSyn—in particular antibodies directed against the transition between NAC domain and the beginning of the CT (dark red color coding for the epitope region; Fig. 2). Certain antibodies directed against CTT aSyn fragments also revealed upregulated immunoreactivities in synucleinopathies, while effects were less pronounced for most antibodies with an N-terminus (NT) epitope (light blue color coding). Interestingly, although the selected AD patients were neuropathologically classified as free of Lewy pathology, the hippocampal detergent-insoluble fractions showed increased immunoreactivities versus controls for several aSyn antibodies as well, including antibodies against CT/CTT aSyn. This could possibly reflect that an early change in aSyn biochemistry preceding apparent LB histopathology, similar to suggestions of previous findings in early PD patients [53].

Together, the observations in the detergent-insoluble fractions indicate an enrichment of various less soluble forms of aSyn in all three brain regions in different neurological conditions compared to age-matched controls. Overall, RPA-derived results suggest differential biochemical manifestations of aSyn levels in brain tissue of patients with different neurological disorders versus controls while cluster analyses further provide indications for disease-specific profiles within the synucleinopathies (Fig. 2).

### Quantification of total aSyn protein levels in human brain tissue extracts using two different immunoassay platforms is robust and validates RPA readout

**Fig. 3:**
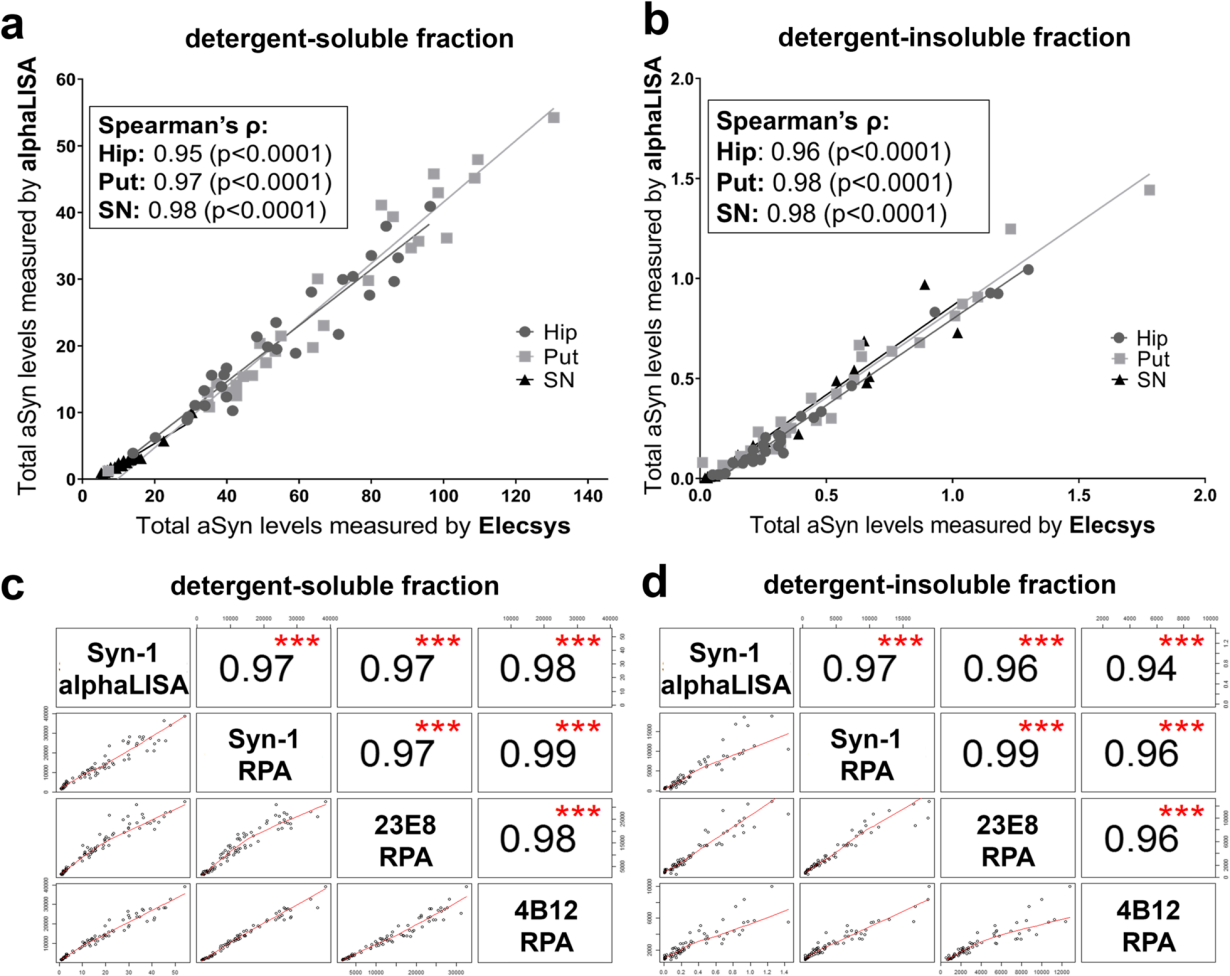
Readout of total aSyn on RPA is validated by two specific immunoassay platforms. **A**, **B**: High correlations between alphaLISA® and Elecsys® platforms in detergent-soluble (**A**) and detergent-insoluble fractions (**B**) in all brain regions. Concentrations on x- and y-axes are both expressed in µg/g wet tissue. Note the substantially lower levels for total aSyn in detergent-soluble fractions of the Substantia nigra (SN) compared to the hippocampus (Hip) and putamen (Put). **C**, **D** quantitative measurements of total aSyn levels as measured by alphaLISA® show high correlations with the RPA signal intensities over all samples, both in detergent-soluble (**C)** and detergent-insoluble (**D**) fractions. Values represent Spearman’s correlation coefficients. ***p < 0.001

To confirm and further explore the observed disease-related immunoreactivity profiles for specific variants of aSyn, including total, CTT, and pSer129 aSyn, we measured their abundance by quantitative immunoassays. For the quantification of total aSyn levels in detergent-soluble and detergent-insoluble fractions of our cohort, we modified the previously published Roche Diagnostics proprietary Elecsys® Total aSyn Prototype Assay [47] and we used it as a reference to validate our newly developed AlphaLISA® assay for total aSyn. Of note, ‘total aSyn’ has to be considered here as ‘putative total aSyn’ as it only detects proteoforms of aSyn that contain or have sterically accessible the epitopes for 23E8 and Syn-1 (i.e., aSyn regions 40–55 and 90–99, respectively). Although certain truncated proteoforms of aSyn lacking these epitopes would count towards the pool of total aSyn, they cannot be detected by this or similar immunoassays for total aSyn. Immunoreactivity detected by 23E8 and Syn-1 on RPA correlated highly with levels for total aSyn on Elecsys® and AlphaLISA® in detergent-soluble and detergent-insoluble fractions of all studied brain regions (Fig. 3). Similarly, total aSyn measured by AlphaLISA® assay also correlated strongly with immunoreactivity on RPA for detergent-soluble and detergent-insoluble tissue fractions for 4B12, an antibody with epitope at res. 103–108 which is near the epitope for Syn-1 (Figs. 1B, 3C, D) [42]. Based on these results, we conclude that semi-quantitative signal intensities measured by RPA reflect the quantitatively measured protein levels obtained by AlphaLISA®, validating our initial approach to analyze total aSyn levels by RPA. In addition, the high comparability between RPA and two immunoassays for total aSyn also indicates that presumably the amounts of aSyn proteoforms containing only one of the epitopes of the antibodies employed in the immunoassays is limited (i.e., such proteoforms would have been detected on the RPA by 23E8 or Syn-1, respectively, but not by Elecsys® and AlphaLISA®, which would have disturbed the correlation between the platforms). Based on this, we conclude that our ‘total aSyn’ measurement likely detects a large proportion of aSyn variants in the brain.

**Fig. 4:**
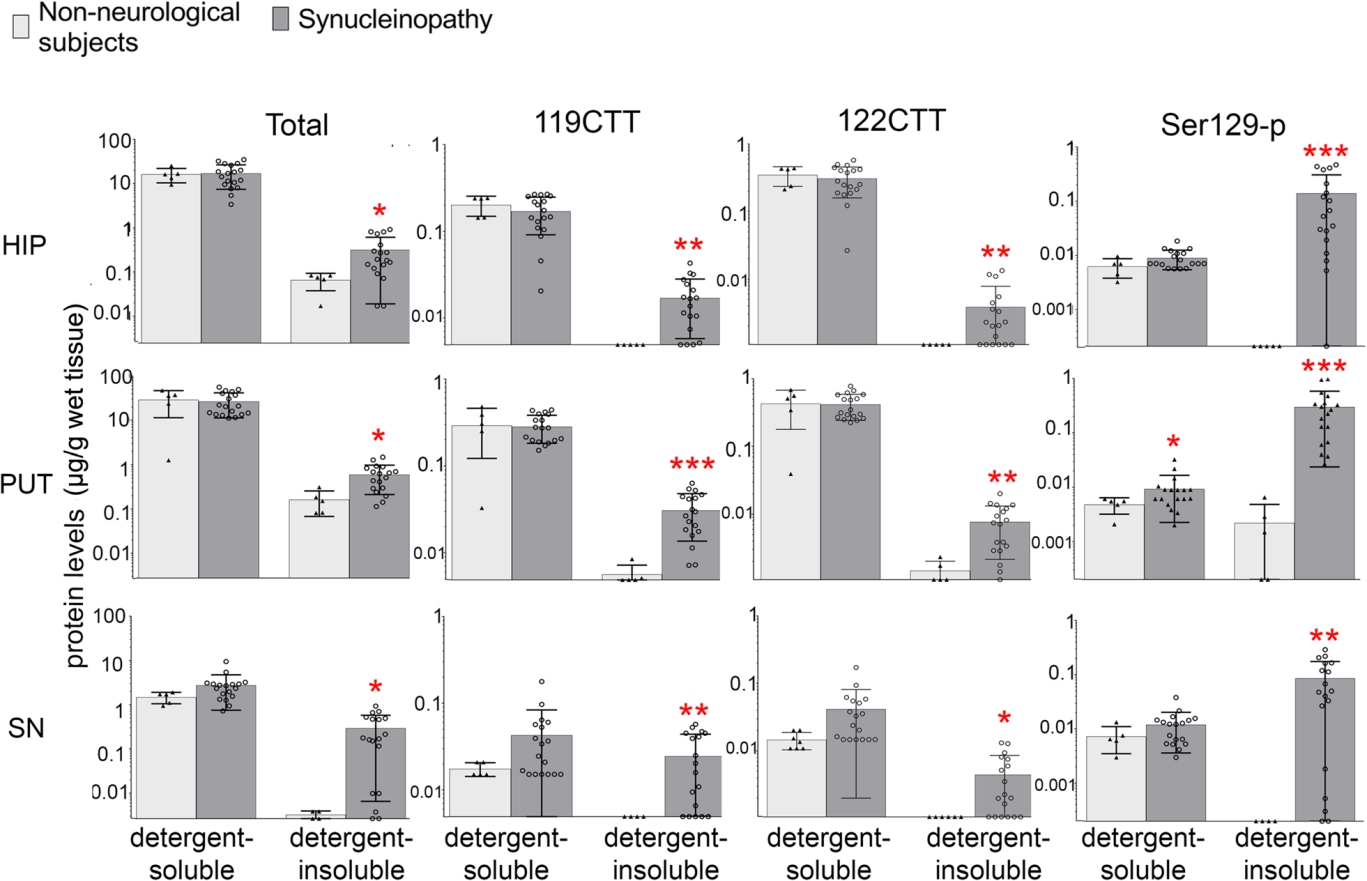
Protein levels for total, 119CTT, 122CTT, and pSer129 aSyn in different fractions of brain tissue extracts of donors with a synucleinopathy and controls. Protein levels are expressed as µg/g wet tissue. Levels of the studied forms of aSyn in brain tissue derived from donors with a synucleinopathy (including PD, PDD, DLB and MSA patients; dark bars) and controls (light bars), in detergent-soluble and –insoluble fractions. Donors with PSP and AD were excluded from the analysis. Statistical comparison between donors with synucleinopathies and controls subjects was done using Mann–Whitney U tests. *p < 0.05; **p < 0.01; ***p < 0.001. Bar graphs indicate mean ± standard deviation

Total aSyn levels were abundantly present in detergent-soluble fractions of all samples, including the different brain regions of all donors, in which we measured a wide range of concentrations (from < 1 until > 50 µg per gram wet tissue). Interestingly, we observed a consistent and marked difference between brain regions using both alphaLISA® and Elecsys®. The total aSyn levels were in average tenfold lower in the SN than in the hippocampus and putamen. This was not reflected by differences in total protein levels between these regions measured by BCA assays (Additional File 1: Fig. S2A). In the RPA analysis, we had also noticed a marked reduced immunoreactivity for detergent-soluble synaptophysin in the SN compared to hippocampus and putamen (Additional File 1: Fig. S2A). In the direct comparison between the RPA immunoreactivities for both presynaptic vesicular membrane-associated proteins aSyn and synaptophysin we observed that the signals positively correlated strongly across all three brain regions (Additional File 1: Fig. S2B). The concomitantly reduced immunoractivity of the presynaptic proteins aSyn and synaptophysin in the SN suggests a lower density in respective synaptic structures in this brain region compared to the other two hence resulting in lower levels of total aSyn as well. On a group level and in any of the analyzed brain regions, quantitative mean levels of detergent-soluble total aSyn as measured by alphaLISA® in donors diagnosed with a synucleinopathy were comparable with the mean levels in controls (Fig. 4) or other analyzed neurodegenerative conditions (Additional File 1: Fig. S3). The variability within the groups was large though, and larger sample sizes may need to be analyzed in the future to discover potential more subtle differences in total aSyn levels between the diagnostic groups.

In contrast to the detergent-soluble total aSyn levels and as expected from the RPA analysis, detergent-insoluble total aSyn levels were significantly elevated in donors diagnosed with a synucleinopathy compared to controls (Fig. 4, *p*-values in HIP/PUT/SN: 0.02/0.007/0.01, respectively). Interestingly, low levels of detergent-insoluble total aSyn were also detected in most of the samples from the individuals with AD, PSP, and controls, who were free of Lewy pathology (Additional File 1: Fig. S3, Table 1). Together, our results indicate that AlphaLISA® is a sensitive and robust method to quantitate total aSyn levels in brain extracts generated by different buffers.

### Detergent-soluble PTM aSyn variants are detectable under physiological and pathological conditions

Immunoreactivity towards 119CTT, 122CTT, and pSer129 aSyn PTM aSyn proteoforms contributed strongly to disease-specific signatures on RPA, especially in the detergent-insoluble fraction (Fig. 2). In order to quantify these aSyn proteoforms, we developed AlphaLISA® assays by pairing the NT-specific 23E8 antibody with the antibodies asyn-131, asyn-134 and asyn-142, respectively (Additional File 1: Table S1, Fig. S1 Fig. S3), for which the selectivity to detect 119CTT, 122CTT, and pSer129 aSyn in human tissue has been described in detail [21].

Levels of PTM aSyn were detectable in the majority of detergent-soluble fractions (yet not in all samples) by AlphaLISA®, both in brain tissue of donors with all analyzed neurological conditions as well as controls (Fig. 4 and Additional File 1: Fig. S3). Intriguingly and similar to total aSyn, levels of 119CTT and 122CTT were by about an order of magnitude higher in the hippocampus and putamen compared to levels in the SN. When considering our measurement of ‘total aSyn’ hypothetically as 100%, values for 119CTT and 122 CTT typically each comprised a fraction of 1–2% of detergent-soluble total aSyn in all analyzed brain regions, with generally slightly higher levels for 122CTT versus 119CTT aSyn in controls (Fig. 4, Additional File 1: Fig. S3). Detergent-soluble pSer129 aSyn concentrations were about 10-times lower than the CTT aSyn levels in the studied brain regions. Again expressed in percent total aSyn, the average proportion of detergent-soluble pSer129 in the hippocampus and putamen of controls was less than 0.1%, while in the SN of controls pSer129 aSyn comprised on average 0.5% of total aSyn. Of note here, although levels for detergent-soluble pSer129 aSyn were above LLD (meaning they are clearly above background and detectable in all detergent-soluble samples), many of them were below the LLOQ. Therefore, quantitative pSer129 aSyn concentrations and relative comparisons with quantitated values of total aSyn have to be used with caution (Additional File 1: Fig. S3).

### The pSer129 aSyn proteoform is largely sequestered into the detergent-insoluble tissue fraction in donors with a synucleinopathy

As in the RPA, detergent-insoluble pSer129, 119CTT and 122CTT levels were detectable by our AlphaLISA® in only in few samples from AD, PSP or controls over all analyzed brain regions (Fig. 2, 4 and Additional File 1: Fig. S4). In contrast, these PTM aSyn proteoforms were detectable in the majority of samples derived from patients diagnosed with a synucleinopathy (Fig. 4 and Additional File 1: Fig. S4). In particular detergent-insoluble pSer129 aSyn was abundantly present under conditions of synucleinopathy, comprising in some samples > 50% of the ‘total’ detergent-insoluble aSyn levels measured. On a group level, detergent-insoluble CTT aSyn species were also enriched in patients with synucleinopathy compared to controls, albeit less pronounced compared to pSer129 aSyn (Fig. 4).

**Fig. 5:**
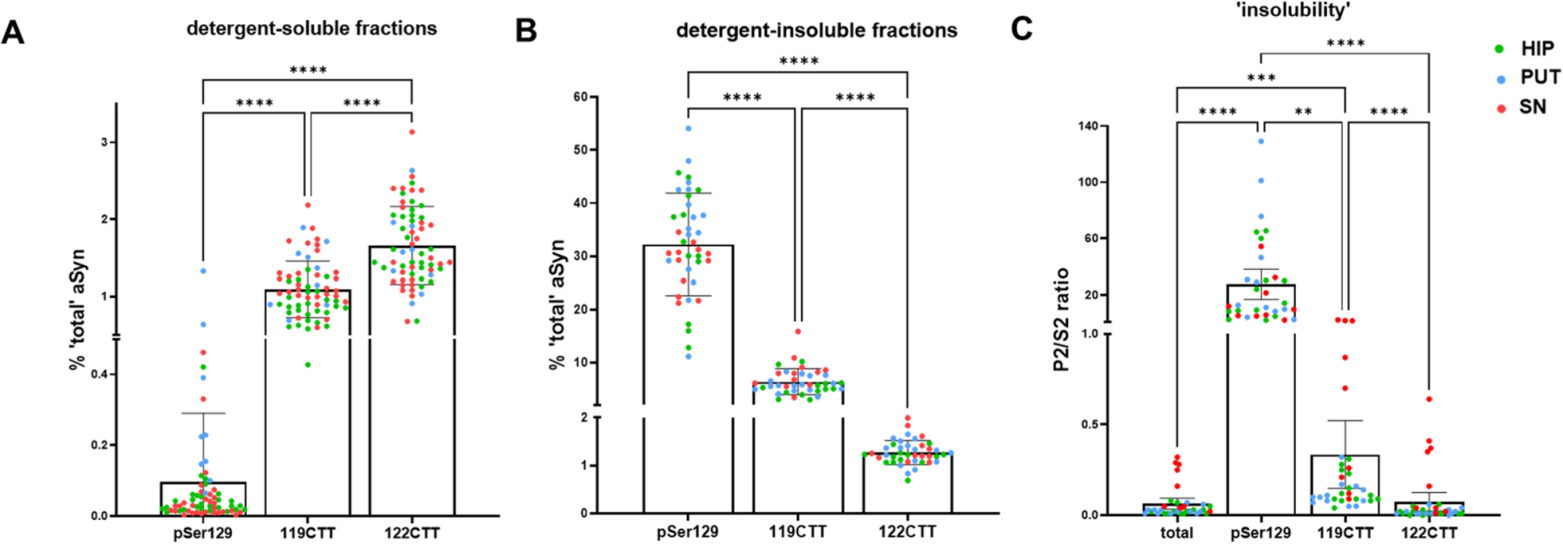
aSyn proteoforms show differential distribution over detergent-soluble and -insoluble fractions. Levels of pSer129, 119CTT and 122CTT aSyn proteoforms expressed as % of putative ‘total’ aSyn levels (see main text) in detectable detergent-soluble (**A**) and -insoluble (**B**) brain fractions included in this study. **C** Distribution of different aSyn proteoforms over sequential fractions, presented as an ‘insolubility’ index by the ratio of protein levels in detergent-insoluble over detergent-soluble fractions of the same tissue specimen. Note: samples were only included in this analysis when all proteoforms were detectable in detergent-soluble/insoluble fractions. Consequently, **A** includes data from patients of all diagnostic groups in our cohort as well as NNS, while **B** and **C** only include data from selected patients with synucleinopathies, as levels of aSyn proteoforms was undetectable in detergent-insoluble fractions of all donors without synucleinopathy and in remaining donors with synucleinopathy. Statistical comparison between different aSyn proteoforms was done using non-parametric Friedman tests and pairwise comparisons using Dunn’s post-hoc tests. *p < 0.05; **p < 0.01; ***p < 0.001, ****p < 0.0001. Bar graphs in **A** and **B** indicate mean ± standard deviation, bar graphs in **C** indicates mean ± 95% confidence interval

The quantitative data in patients with a synucleinopathy also help to estimate relative differences between protein variants in biochemical sequestration. For instance, levels of detergent-insoluble 119CTT aSyn were generally higher (up to 10% of detergent-insoluble total aSyn levels) than for 122CTT aSyn (up to 2% of detergent-insoluble total aSyn levels; Figs. 4, 5). More strikingly, while levels of total, 119CTT and 122CTT aSyn in donors with a synucleinopathy were generally lower in the detergent-insoluble compared to the detergent-soluble fraction levels, pSer129 aSyn levels were > tenfold higher in detergent-insoluble versus detergent-soluble fractions (Figs. 4, 5), quantitatively confirming previous results that the large majority of pSer129 aSyn is sequestered into detergent-insoluble fractions under pathological conditions [18].

### Biochemical sequestration of neurodegenerative disorders based on quantitative total and PTM aSyn levels in different brain regions

**Fig. 6:**
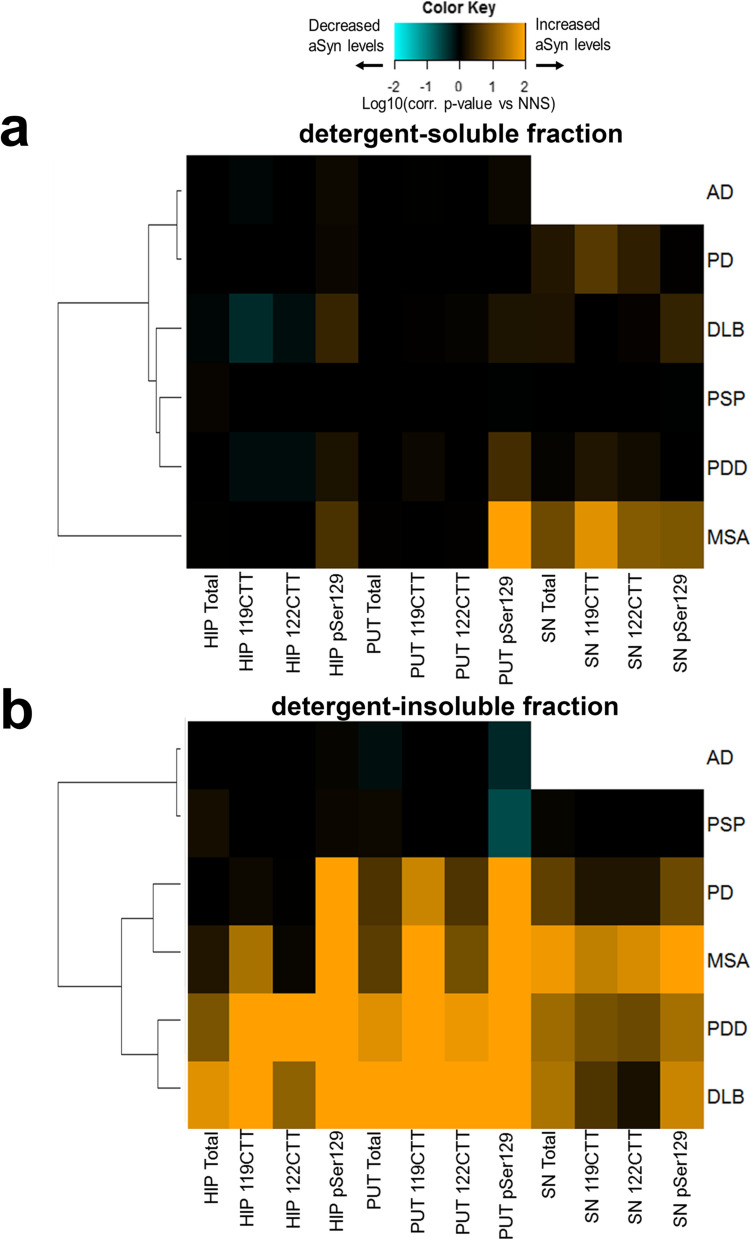
Overview of total, pSer129, 119CTT, and 122CTT aSyn levels in different synucleinopathies and tauopathies as compared to control subjects. Overview of log10-transformed p-values of group comparisons in detergent-soluble (**A**) and -insoluble (**B**) fractions after Dunnett’s post-hoc correction. Colors indicate the significance level for each diagnostic group compared to controls. Note the SN of the AD patients was not processed. HIP = hippocampus; PUT = putamen; SN = substantia nigra

Similar to the RPA analysis, we now asked whether patient groups with distinct neurological disorders display specific biochemical signatures based on quantitative measures of total and PTM aSyn. The individual data points for all measurements are shown in Additional File 1: Fig. S3 (detergent-soluble fractions) and Additional File 1: Fig. S4 (detergent-insoluble fractions), while p-values resulting from the comparison of protein levels for each diagnostic group versus controls were log10-transformed and visualized in hierarchically clustered heat maps as done for the RPA data (Fig. 6).

In detergent-soluble fractions, MSA stood out from the other disorders, despite the smaller sample size (Additional File 1: Fig. S3; Table 1, N = 3 for MSA versus N = 5 for controls and the other synucleinopathies). In particular, the putamen of MSA patients compared to controls showed strongly elevated levels of detergent-soluble pSer129 aSyn (mean ± SD controls/MSA: 0.004 ± 0.001/0.018 ± 0.009 µg/g wet tissue; p = 0.004) but to lesser extent for 119CTT and 122CTT aSyn. In the SN, detergent-soluble 119CTT aSyn levels were significantly elevated in MSA patients (mean ± SD controls/MSA: 0.017 ± 0.003/0.073 ± 0.02 µg/g wet tissue; p = 0.016) while the elevated average levels of pSer129 and 122CTT aSyn proteoforms did not reach significance (Additional File 1: Fig. S2; Fig. 6A). Still, the higher levels of detergent-soluble aSyn proteoforms in the SN of MSA patients replicated the increased immunoreactivities observed by RPA in the same extract (Fig. 2).

Also in line with the findings by RPA (Fig. 2), the levels of detergent-insoluble PTM aSyn proteoforms were strikingly increased in the hippocampus and putamen of PDD and DLB patients compared to controls (Additional File 1: Fig. S4, Fig. 6B). Detergent-insoluble PTM aSyn variants were not detectable in the vast majority of samples from controls, AD and PSP donors (Additional File 1: Fig. S4). Differences in donors with synucleinopathies compared to controls were most pronounced for pSer129 aSyn (mean ± SD controls/PDD/DLB in the hippocampus: < LLD (0.0002)/0.20 ± 0.18/0.22 ± 0.15 µg/g wet tissue; mean ± SD controls/PDD/DLB in the putamen: 0.002 ± 0.002/0.27 ± 0.24/0.37 ± 0.21 µg/g wet tissue; p < 0.001, Additional File 1: Fig. S4). Interestingly, in the hippocampus, average pSer129 aSyn levels were substantially higher in PDD and DLB patients as compared to PD patients without dementia in this study (Additional File 1: Fig. S4), despite similar Braak LB stage (6) in these donors (Table 1). Detergent-insoluble 119CTT and 122CTT levels followed the same trend albeit less pronounced than for pSer129 aSyn. In the SN, most significant increases compared to controls were observed for detergent-insoluble total and PTM aSyn levels in MSA patients (Fig. 6B), similar to our previous observation in RPAs. Notably, within-group variabilities were generally larger in the SN than in hippocampus and putamen (Additional File 1: Fig. S4).

In further support of a specific signature for the biochemical sequestration of aSyn proteoforms, unsupervised hierarchical clustering of p-values for each diagnostic group in comparison to levels detected in controls revealed separation of MSA patients from the other diagnostic groups in the detergent-soluble fraction of the putamen and SN (Fig. 6A). Based on differences versus controls in detergent-insoluble fractions (Fig. 6B), synucleinopathies clustered separately from AD and PSP. Not quite unexpected, PDD and DLB patients co-clustered in the second degree of the dendrogram, based on highest increased levels of detergent-insoluble aSyn proteoforms in the hippocampus and putamen. In contrast, PD and MSA co-clustered on a separate second-degree cluster, due to the similar p-values obtained for detergent-insoluble pSer129 in hippocampus and putamen and 119CTT in putamen, respectively.

Together, although in a restricted sample size, our quantitative results measured by sandwich type immunoassays confirm and validate the semi-quantitative RPA results, suggesting differential biochemical aSyn signatures between neurodegenerative diseases and brain regions, and specifically within synucleinopathies.

## Discussion

aSyn is one of the most abundant proteins in the entire nervous system (extensively reviewed in [54]), and is neuropathologically and in some rare cases also genetically at the center of synucleinopathies such as PD(D), DLB, and MSA [2, 4, 55, 56]. aSyn pathology is characterized as neuronal or glial accumulations of aSyn inclusions like LBs or GCIs and it develops in a progressive and currently unstoppable manner over many decades throughout various brain regions, spinal cord and peripheral tissues [57]. Although the mechanistic link is still unclear, the progression of the pathology affects motor and non-motor functions leading to debilitating and continuously worsening neurological conditions. Only recently, clinical trials have started to target aSyn by certain aSyn-specific antibodies to test their therapeutic potential in PD [58–60]. At this point, however, very little is known about quantitative biochemical proportions of different proteoforms of aSyn in the human brain tissue. Having such information may support a better understanding of the role of aSyn in the pathogenesis of synucleinopathies and also potentially help to identify novel biomarkers of disease progression and diagnostic tools to distinguish between different synucleinopathies. Additionally, such information is needed for optimally tailoring novel therapies in personalized healthcare approaches, e.g. for modeling pharmacological relationships between aSyn and molecules which target this protein or its genetic information in respective patient populations with different synucleinopathies or at different stages of the disease.

In this study, we aimed for quantification of different aSyn proteoforms, including total, 119CTT, 122CTT, and pSer129 aSyn, in the human brain under physiological and various neuropathological conditions. For this, we used fractionated tissue extracts of three different brain regions from clinically and neuropathologically well characterized individuals. Our study includes samples from six different neurological conditions, of which four were confirmed synucleinopathies, and from a control group of aged individuals who had no records of neurological symptoms during life. Employing first a semi-quantitative multiplex approach with 65 aSyn-specific antibodies by RPA (e.g., in essence this is a fractionated brain lysate-based protein array) [46, 52] followed by multiple quantitative immunoassays for the different aSyn proteoforms and epitopes, we tested our hypothesis that different patient groups would show disease-specific biochemical profiles of aSyn protein variants in the brain.

Our quantitative analysis in the human brain showed a marked increase of total, pSer129 aSyn and CTT aSyn in detergent-insoluble fractions of brain tissue samples from individuals with a synucleinopathy compared to controls, which is in line with previous (semi-)quantitative studies by others [16, 18, 23–26, 61]. This supports the observation in PD brain by super-resolution microscopy of the accumulation and sequestering of these PTMs in cellular compartments that are suggested to be detergent-insoluble during biochemical processing of the tissue [21]. Moreover, although in a restricted sample size, we observed differences in the biochemical aSyn profiles between patient groups with distinct synucleinopathies, suggesting disease-specific profiles that may provide several novel insights into the underlying pathophysiology of these diseases. For instance, MSA samples clustered separately from other synucleinopathies based on their patterns of increased aSyn variants compared to controls (Fig. 6). These patients mainly stood out because of increased levels of detergent-soluble aSyn species in the SN, and pSer129 aSyn in the putamen compared to controls (Figs. 2, 6), which is in line with a study that employed semi-quantitative analyses of Western blots in brain tissue from PD, PDD and MSA [62]. Whether this is specific to the subjects analyzed in this study, needs to be explored in brain tissue of additional MSA donors. Nevertheless, the differentiation of MSA from the other synucleinopathies corroborates recent findings that pathological aSyn in GCIs may be biochemically, structurally and biologically distinct from aSyn in LBs [22, 63]. Another intriguing observation in MSA patients was that differences versus controls were more pronounced in detergent-insoluble extracts of the SN compared to hippocampus and the putamen, despite extensive GCI pathology in the latter region. Thus, although both putamen and SN display GCI pathology, the aSyn biochemical signature in the SN revealed many more differences compared to the normal-aged brain particularly by RPA. The limited differences in insoluble aSyn species in a region with substantial GCI pathology is in support with results from a previous study, which showed a poor correlation between GCI pathology and detergent-insoluble aSyn over different brain regions [64] while another study previously reported pronounced histopathological changes beyond GCI pathology in the SN and other regions of MSA-P donors [65]. Thus, our findings support that aSyn pathology in MSA has a different biochemical signature compared to diseases characterized by LB pathology (PD/PDD/DLB). Although this could potentially reflect different biochemical composition of pathological hallmark lesions (GCI versus LBs), in our biochemical study design we are not able to address the expression and accumulation of aSyn in different cell types which is routinely studied in context of synucleinopathies using immunostainings [66–68]. Our results exemplified in MSA together with the reports in literature highlight, that immunostainings and biochemical approaches can provide complementary information in the phenotyping of aSyn pathology, and their combined use may allow for better characterization and differentiation of synucleinopathies.

We further observed that, perhaps not surprisingly, PDD and DLB patients co-clustered together in our RPA and AlphaLISA® analyses based on profound enrichment of detergent-insoluble aSyn PTM proteoforms in the hippocampus and putamen compared to controls (Fig. 6). Detergent-insoluble pSer129 aSyn levels in the hippocampus were significantly higher in PDD and DLB patients compared to PD patients, while other detergent-insoluble aSyn variants followed the same trend. This finding is in line with the results of a previous study showing increased hippocampal SDS-soluble pSer129 and total aSyn levels in PDD compared to PD [23]. Further, immunostaining-based reports have linked the occurrence of dementia in PD to an increased local load of hippocampal and cortical Lewy pathology [69–72]. In our study, increased detergent-insoluble aSyn variants in PDD/DLB patients compared to PD patients were not explained by differences in regional distribution of LBs (e.g. Braak LB stages) or disease duration, which were similar between these patient groups. This may suggest that a higher regional load of insoluble aSyn in the hippocampus of PDD and DLB patients could represent a pathological correlate for dementia in these diseases, which is less well reflected by the overall distribution of LB pathology as used in the Braak staging. While our study in small patient groups did not address the relationship between insoluble (PTM) aSyn variants in hippocampal and cortical regions with clinical scales for dementia, a recent study reported correlations between pSer129 aSyn concentrations in neo-cortical brain detergent-insoluble extracts of 15 patients with LB-dementia (PDD/DLB) measured by ELISA and worse score on scales for Clinical Dementia Rating and MMSE during life [27]. Future studies containing larger numbers of clinically well-characterized donors should further explore the relation of insoluble PTM aSyn levels and occurrence/severity of dementia by different clinical scales in PD/DLB. Doing so may help shedding more light onto mechanistic links between aSyn pathology or the biochemical signatures of aSyn with functional losses in the different disease processes.

Although our study was done using a relative limited sample size (n = 28 individuals in total), the strength of this study is the systematic analysis of different aSyn proteoforms in different regions of carefully selected samples based on extensive neuropathological characterization and limited concomitant pathology using various assays. Additionally, the overall changes in aSyn species are very robust over multiple assay platforms which employed state-of-the art generated and validated epitope and PTM-specific antibodies. Importantly, the quantitative alphaLISA® enabled the calculation of the percent distribution of different proteoforms of aSyn in comparison to a ‘total aSyn’ measure within in the same tissue fraction, which will be helpful for the comparison with future such studies (Fig. 5). We noted that abundance of fractionated pSer129 aSyn would possibly be sufficient to differentiate synucleinopathies from each other or from other neurodegenerative conditions (Fig. 6). Yet, the combination of multiple antibodies in the RPA and the quantitative immunoassays allowed for much better recognition of similarities and subtle differences between synucleinopathies, e.g. by co-cluster analysis. It will be interesting to explore how the signatures of aSyn proteoforms can be strengthened and further validated in larger sample of clinically-well-defined cohorts including carriers of a genetic risk to develop PD as well as sporadic PD patients.

Our measurements of detergent-soluble total, 119CTT and 122CTT aSyn levels demonstrated large differences between the studied brain regions, as substantially lower protein levels were measured for these variants in the SN as compared to hippocampus and putamen (Additional File 1: Fig. S3). This is in line with a previous study performed by Western blot [73]. Noteworthy, we previously showed that the *SNCA* mRNA levels are lower in the SN than in the cortical regions using microarray and RNA sequencing datasets [74]. The reported gene-expression modules (including *SNCA*) that correlated positively with Braak LB stages (aSyn immunostaining of Lewy pathology) were specifically enriched for neuronal markers and related functions. Altogether, these data underscored the large regional differences in neuronal aSyn expression, with lowest aSyn levels in the SN and highest expression in limbic and neocortical brain regions. We show in this biochemical study here that such regional differences were not accompanied by reduction of total protein levels (Additional File 1: Fig. S2A). Consistent with the published gene expression data, RPA signal intensities for the synaptic marker synaptophysin were substantially lower in the SN compared to the putamen and hippocampus and they correlated strongly with aSyn (Syn-1) across all analyzed brain regions (Additional File 1: Fig. S2B). This finding suggests that lower levels of (total) aSyn and synaptophysin in the SN reflects a lower density of synapses in this region, as both proteins are mainly enriched at presynaptic terminals under physiological conditions. A lower synaptic density in the midbrain compared to putamen and hippocampus was suggested before by microscopic and biochemical mapping of the expression of the synaptic markers SAP102 and PSD95 in mouse brains [75, 76]. In support of this in humans, in vivo positron emission tomography (PET) imaging of a synaptic vesicle glycoprotein 2A radioligand in the brain of healthy subjects showed reduced binding potential in the midbrain as compared to hippocampus and putamen [77].

CTT variants of aSyn or pSer129 aSyn have been associated with Lewy pathology [17, 18, 20, 21] and thus, due to the proposed reduced solubility of proteins in Lewy pathology, these proteoforms were expected mainly in detergent-insoluble tissue fractions. It was therefore remarkable to detect these proteoforms not only in synucleinopathies, but also in the detergent-soluble fraction of neurologically normal aged subjects (controls) and neurological cases without a synucleinopathy (e.g. AD and PSP) (Figs. 2, 6, Additional File 1: Fig. S3). We had excluded SDS from RIPA, which is known to be suboptimal for solubilization of proteins in tissue extract. The reason to omit SDS was, however, to avoid any unwanted interference of aSyn with SDS micelles and nuclear DNA [33–37]. This omission of SDS in our study may also lead to a segregation of certain aSyn proteoforms in detergent-soluble versus detergent-insoluble fractions that is different from what is reported in studies where SDS was employed. However comparison of results between different studies is generally difficult as also detergents other than SDS were used (e.g., Triton X-100) or also in some cases heat stable tissue extracts were generated that may lead to precipitation of some aSyn proteoforms (for a comparison of different methods see for instance [15–22]). By applying an ultracentrifugation step on our mild detergent SDS-free RIPA extract (Fig. 1A) we sedimented presumably all insoluble species of proteins from the detergent-soluble fraction. In addition, the clear segregation of the three neuronal markers NeuN, NSE and synaptophysin to their anticipated fractions (Additional File 1: Fig. S2) supports the robustness of our fractionation protocol and indicates that our observation is much less likely to be an artifact of sample preparation (e.g. that a small fraction of the presumed detergent-insoluble aSyn from Lewy pathology has ended up in the detergent-soluble fraction). Thus, the presence of CTT and pSer129 aSyn in the detergent-soluble fraction of our mild fractionation protocol points to a potential relevant physiological process that generates CTT and pSer129 at low levels, supporting previous suggestions by others [16, 18, 73]. The detection of 122CTT aSyn under conditions of normal aging is further supported by our recent study in which we demonstrate localization of 122CTT aSyn to the outer membrane of mitochondria in the brain of aged neurologically normal subjects by STED microscopy [21].

Of note, CTT variants of aSyn and pSer129 aSyn have been associated with Lewy pathology in the human brain because they are highly enriched in these pathological features when analyzing immunostainings by microscopy [17, 18, 20, 21]. In addition, pSer129 and CTT aSyn segregate biochemically into the SDS-insoluble fractions and subsequent analysis by Western blot favors the detection of these high abundant proteins in synucleinopathies, while not being sensitive enough for low abundant (physiological) soluble proteoforms of aSyn. Our approach with RPA remains more robust for the detection of low abundant proteoforms in the detergent-soluble fraction due to an enrichment of proteins in one spot on the array and detection by immunofluorescence, which has a higher sensitivity than the typical Western blot [46]. In addition, immunoassays such as alphaLISA® designed with highly selective and specific antibodies (Additional File 1: Table S1, Fig. S1; [21]) allow for the reliable quantification of low abundant proteoforms of aSyn also in the detergent-soluble fraction (Fig. S3).

Although phosphorylation at Ser129 is widely considered to be mainly a pathology-associated modification of aSyn, we also detected low amounts (< 1% of total aSyn) of soluble pSer129 aSyn in non-diseased brain (Figs. 4, 5 and Additional File 1: S3). This is in line with previous findings by quantitative [23, 24], and semi-quantitative [18, 51, 73] approaches. It is noteworthy, that steady-state detection of pSer129 aSyn in postmortem brain tissue (as also performed in our study) likely underestimate the importance of pSer129 aSyn in physiological conditions because of the dynamic nature of this reversible PTM [78]. The stability of detergent-soluble pSer129 aSyn (or other phosphorylated proteins) in postmortem tissue is at risk due to rapid dephosphorylation or other protein destabilizing processes that are known to occur in tissue shortly after death [79–81]. In line with a physiological role of pSer129 aSyn, mechanistic studies have attributed various functions to pSer129 aSyn (for instance by introducing Ser129Ala mutations) such as regulating the proposed nuclear localization of aSyn [82], proteolytic degradation [83, 84], and the cellular uptake of dopamine [85].

One possible explanation for the increased abundance of PTM aSyn variants such as CTT aSyn under conditions of synucleinopathy may be that certain enzymes like calpains [86, 87] and caspase-1 [88] are processing aSyn in the accumulated inclusion bodies. Such cleavage and phosphorylation of aSyn may also be actively regulated, as a recent study suggested based on a cellular model of aSyn fibril-induced inclusion bodies [89]. Also phosphorylation of aSyn at Ser129 and C-terminal truncation has been proposed for rendering aSyn less soluble [51, 90]. However, as limited correlation was previously found between detergent-insoluble aSyn species and Lewy pathology in the brain of PD and DLB patients, it has been suggested that the intracellular insoluble aSyn species are formed before their sequestration in LBs [24, 53, 91]. Clearly, pSer129 aSyn is located in what appears to be an outer shell of Lewy bodies but in addition granular or reticular pSer129 aSyn immunoreactivity is observed in the cytoplasm of neurons in the PD brain, possibly representing an early stage of aSyn cytopathology [21]. Moreover, recent correlative light and electron microscopy studies indicate that aSyn in Lewy bodies and neurites seems to be trapped between distorted membrane fragments, organelles and vesicles [92]. Due to the robust binding of aSyn to membranes it is conceivable this leads to a biochemical sequestration of different forms of aSyn and strong detergents or solvents may be required to extract aSyn from this highly lipid-enriched environment. Importantly, the detergents should not interfere negatively with aggregation state of aSyn, which is an issue with SDS [34].

In summary, here we provide novel insights into the quantitative abundance of different aSyn proteoforms and their biochemical distribution in the brain of aged neurologically normal individuals, donors with different synucleinopathies and other neurodegenerative conditions. Results of our correlative approach between different antibody-based platforms suggested quantitative differences in biochemical profiles for aSyn proteoforms among different brain regions and synucleinopathies. This finding corroborates previous (semi-)quantitative reports and provides the field with currently missing quantitative readouts of aSyn proteoforms in the human brain. The results can hopefully contribute to development of better translational models of synucleinopathies and enable exploration of biochemical disease-specific tissue biomarkers to distinguish different synucleinopathies in personalized healthcare approaches and for providing further rationale and context to develop novel aSyn-related therapies.

## Supplementary Information

**Additional file 1.** Supplementary tables and figures: **Table S1**: AlphaLISA® antibody specifics; **Table S2**: RPA antibody specifics. **Fig. S1**: AlphaLISA® hookpoints & standard curves. **Fig. S2**: Total protein & RPA neuronal and synaptic markers. **Fig. S3**: AlphaLISA® quantification of detergent-soluble fractions. **Fig. S4**: AlphaLISA® quantification of detergent-insoluble fractions

## Data Availability

The data that support the findings of this study are available from the corresponding author M.B. upon reasonable request.
