# Supplementary material for: Multi-platform quantitation of alpha-synuclein human brain proteoforms suggests disease-specific biochemical profiles of synucleinopathies
Source: Acta Neuropathol Commun. 2022 Jun 3;10:82. doi: 10.1186/s40478-022-01382-z (PMC9164351; doi:10.1186/s40478-022-01382-z)
Supplement: Supplementary file 1 — Additional file 1. Supplementary tables and figures: Table S1: AlphaLISA® antibody specifics; Table S2: RPA antibody specifics. Fig. S1: AlphaLISA® hookpoints & standard curves. Fig. S2: Total protein & RPA neuronal and synaptic markers. Fig. S3: AlphaLISA® quantification of detergent-soluble fractions. Fig. S4: AlphaLISA® quantification of detergent-insoluble fractions [file 40478_2022_1382_MOESM1_ESM.docx]

**Supplementary Tables and Figures**

**Table S1: Antibody specifics for alphaLISA® assays for total and post-translationally modified forms of aSyn**

| **Targeted aSyn species** | **Acceptor beads-conjugated antibody (µg/ml)** | **Epitope**  **(*Source*)** | **LLD in well (ng/ml)** | **LLOQ in well (ng/ml)** |
| --- | --- | --- | --- | --- |
| Total aSyn | Syn-1  (10) | res 91-99  *(BD biosciences)* | 0.02 | 0.05 |
| 119CTT | Syn-131  (10) | CTT119-specific  *(Roche)* | 0.02 | 0.046 |
| 122CTT | Syn-134  (10) | CTT122-specific  *(Roche*) | 0.01 | 0.014 |
| pSer129 | Syn-142  (10) | pSer129-specific  *(Roche)* | 0.002 | 0.006 |

For the respective quantification of the targeted aSyn species, antibody 23E8 (aSyn epitope region amino acid 40-55; Prothena, South San Francisco, CA, USA) was paired in an alphaLISA® assay with the mAbs indicated above. For specifics about assay setup see methods. LLD, lower limit of detection; LLOQ, lower limit of quantification.

**Table S2: Summary table for the antibodies included in reverse array assays**

| **AB** | **Epitope or epitope region** | **Source** | **Species**** |
| --- | --- | --- | --- |
| Clone 42 | 91-99 (NAC) | BD Transduction Laboratories (#610787) | Mouse |
| 4B12 | 103-108 CT) | Covance (#SIG-39730) | Mouse |
| 15G7 | 116-131 (CT) | Enzo LifeScience | Rat |
| 211 | 121-125 (CT) | Santa Cruz (#sc-12767) | Mouse |
| 23E8 | 40-55 (NT) | Prothena | Mouse |
| 9A6 | 91-99 (NAC/CT) | Prothena | Mouse |
| 9G5 | 91-99 (NAC/CT) | Prothena | Mouse |
| 1H7 | 91-99 (NAC/CT) | Prothena | Mouse |
| 5C12 | 111-118 (CT) | Prothena | Mouse |
| 5C1 | 118-126 (CT) | Prothena | Mouse |
| 8A5 | 125–140 (CT) | Prothena | Mouse |
| 7H2 | aa122 (122CTT | Prothena | Mouse |
| syn105 | aa122 (122CTT)* | Prothena | Rabbit |
| 11A5 | Ser129p | Prothena | Mouse |
| 14 different Mabs | NT | Roche | Rabbit |
| 2 different Mabs | 25-43 (NT) | Roche | Rabbit |
| 1 Mab | 45-60 (NT) | Roche | Rabbit |
| 3 different Mabs | 41-63 (NT) | Roche | Rabbit |
| 2 Mabs | NAC | Roche | Rabbit |
| 1 Mab | 93-107 (NAC/CT) | Roche | Rabbit |
| 1 Mab | 87-119 (NAC/CT) | Roche | Rabbit |
| 7 different Mabs | 96-140 (CT) | Roche | Rabbit |
| 1 Mab | 97-114 (CT) | Roche | Rabbit |
| 3 different Mabs | aa119 (119CTT) | Roche | Rabbit |
| 2 different Mabs | aa122 (122CTT) | Roche | Rabbit |
| 4 different Mabs | pSer129 | Roche | Rabbit |
| 10 different Mabs | Recombinant human aSyn1-140 | Roche | Rabbit |
| Neuron-specific enolase (NSE) | Not available | Millipore (AB951-l) | Rabbit (polyclonal) |
| Neuronal Nuclei (NeuN) clone A60 | Not available | Millipore (MAB377) | Mouse |
| Synaptophysin, clone SY38 | Not available | Millipore (Mab5258-50UG) | Mouse |

* syn105 strongly binds aSyn truncated at aa122 but also shows some cross-reactivity to aa121 and aa123 aSyn (unpublished data by Prothena)

** Antibodies were all monoclonal, if not indicated differently

aa, amino acid; AB, antibody; CT, C-terminal; CTT. C-terminally truncated; Mabs, monoclonal antibodies; NAC, non-β-amyloid component of Alzheimer’s plaques; NT, N-terminal; pSer129, phosphorylated at Serine 129

**
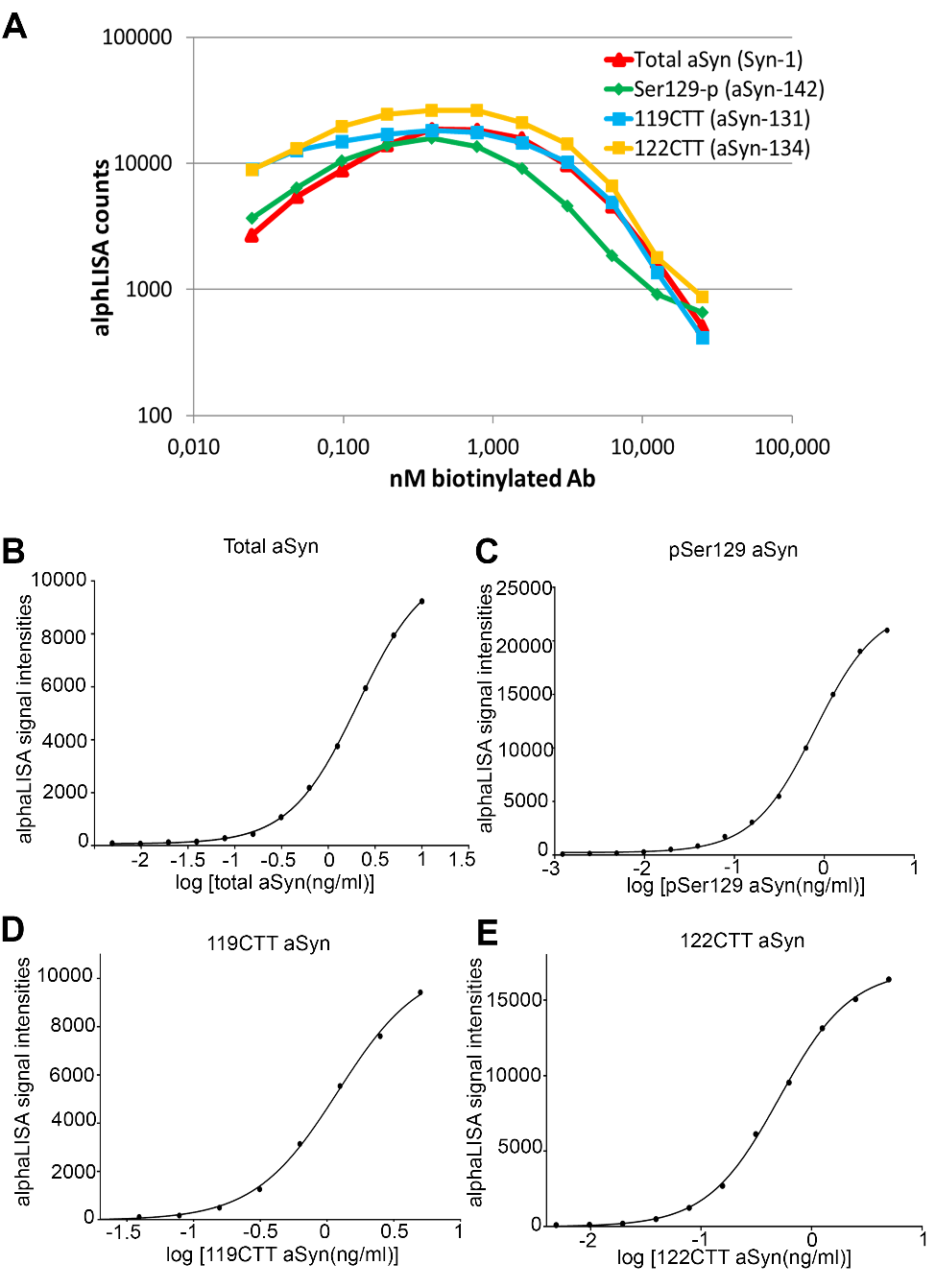
**

**Figure S1: Hook points and standard curves for alphaLISA® assays for the quantitative measurement of total and PTM aSyn. (A)** Results from hook point analysis for the developed alphaLISA**®** assays. Assays for total, CTT and pSer129 aSyn were developed using the appropriate aSyn species in a concentration of 5µg/ml and acceptor beads in a concentration of 10µg/ml. Based on the hook points, the concentration of biotinylated 23E8 was established at 0.3 nM for pSer129 aSyn assays and 0.8 nM for Total, 119CTT and 122CTT aSyn assays. (**B-D)** Standard curves for the developed alphaLISA**®** assays for the quantitative measurement of total (**B**), pSer129 (**C**), 119CTT (**D**), and 122CTT (**E**) aSyn.


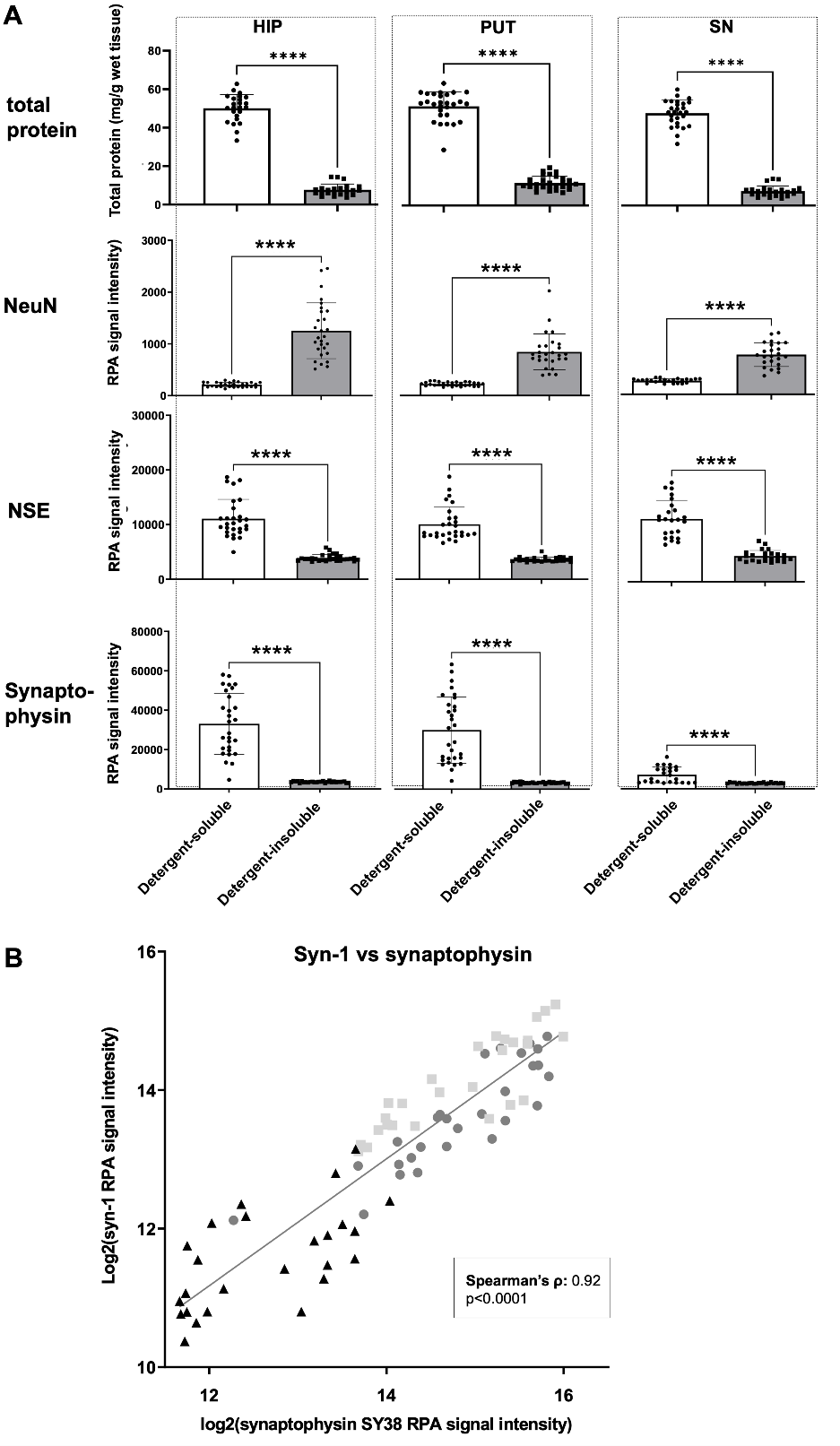


**Figure S2: Biochemical and regional distribution of total protein and RPA signal intensities for neuronal and synaptic markers. (A)** Distribution of total protein levels measured by BCA and RPA signal intensities (immunofluorescence) for neuronal nuclei (NeuN), neuron-specific enolase (NSE), and synaptophysin in detergent-soluble and -insoluble fractions per brain region. Note the decreased RPA signal intensities for synaptophysin in the soluble fractions of the SN compared to hippocampus and putamen. HIP: hippocampus; PUT: putamen; SN: substantia nigra. Statistical comparison between different detergent-soluble and -insoluble fractions: Student t-test. ****, p<0.0001. **(B)** Scatter plot and trend line of a linear regression analysis for log2-normalized RPA signal intensities of syn-1 (aSyn) versus SY38 (synaptophysin) antibody in detergent soluble fractions. Note the overall good association of immunoreactivity towards the two presynaptic vesicular membrane proteins aSyn and synaptophysin in all brain regions and the concomitant low signals for both proteins in SN compared to the two other brain regions. Spearman’s rank correlation coefficient was calculated and *p*-value determined.

**
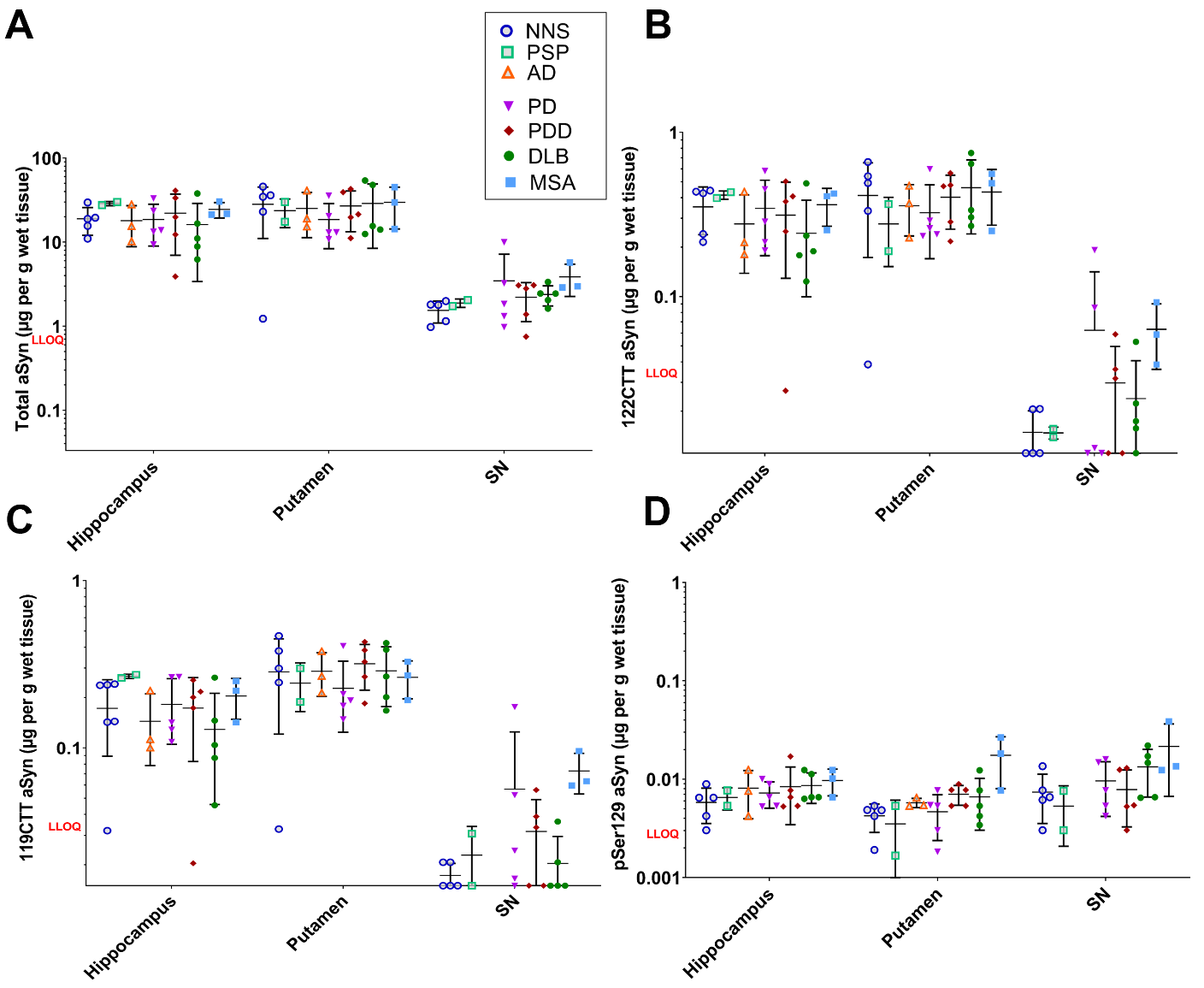
Figure S3:** **Quantification of detergent-soluble fractions.** Mean levels (±SD) of the studied forms of total (**A)**, 119CTT (**B**), 122CTT (**C**) and pSer129 (**D**) aSyn in detergent-soluble tissue fractions per brain region and diagnostic group. The baseline of the y-axis is set at the lower limit of detection (LLD) of each assay, while the lower limit of quantification (LLOQ) is indicated. Note, SN of AD was not processed.

**
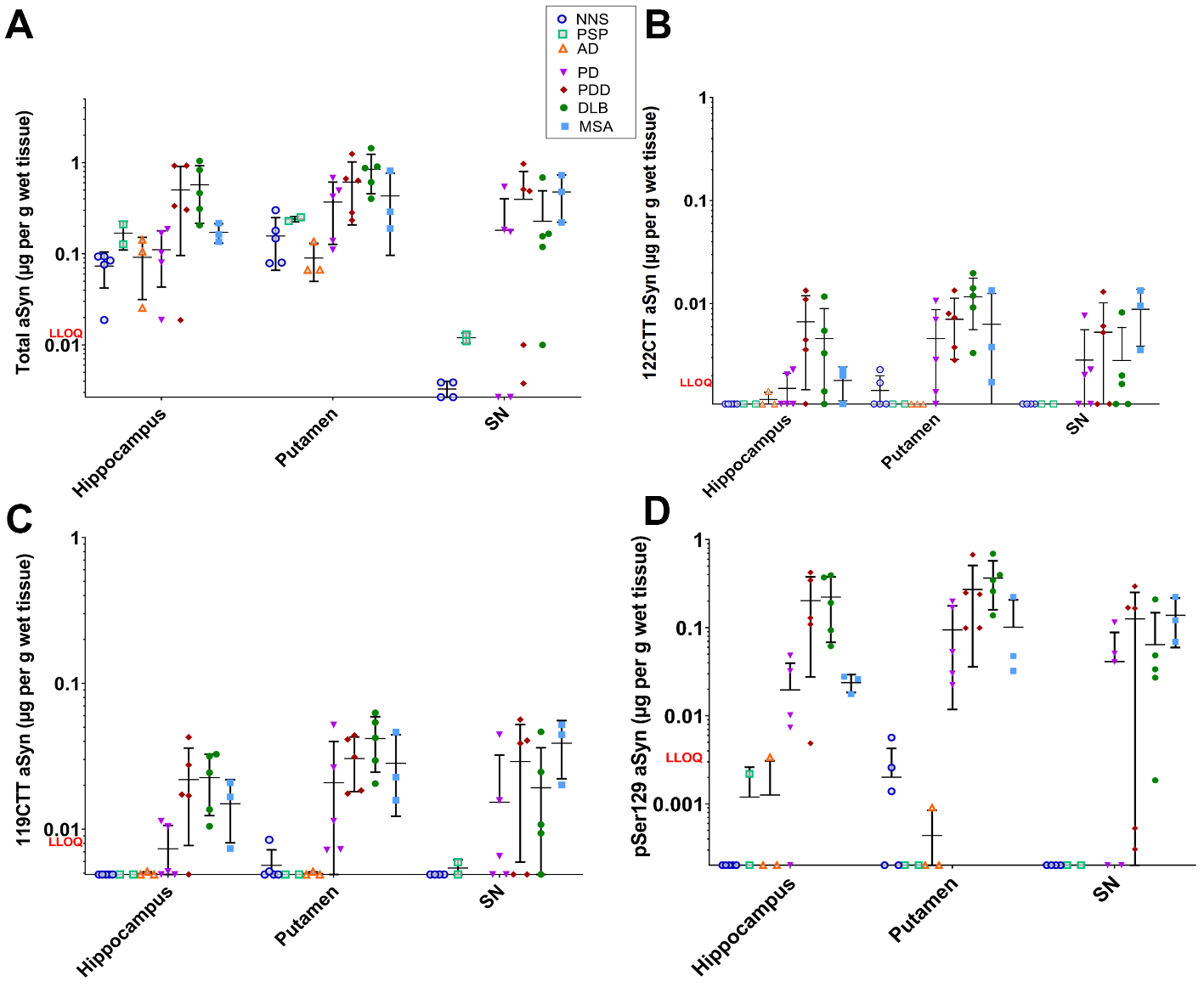
**

**Figure S4: Quantification of detergent-insoluble fractions.** Mean levels (±SD) of the studied forms of total (**A)**, 119CTT (**B**), 122CTT (**C**) and pSer129 (**D**) aSyn in detergent-insoluble tissue fractions per brain region and diagnostic group. The baseline of the y-axis is set at the lower limit of detection (LLD) of each assay, while the lower limit of quantification (LLOQ) is indicated. Note: SN of AD was not processed.
